# Polymicrobial sepsis influences NK-cell-mediated immunity by diminishing NK-cell-intrinsic receptor-mediated effector responses to viral ligands or infections

**DOI:** 10.1371/journal.ppat.1007405

**Published:** 2018-10-31

**Authors:** Isaac J. Jensen, Christina S. Winborn, Micaela G. Fosdick, Peng Shao, Mikaela M. Tremblay, Qiang Shan, Sandeep Kumar Tripathy, Christopher M. Snyder, Hai-Hui Xue, Thomas S. Griffith, Jon C. Houtman, Vladimir P. Badovinac

**Affiliations:** 1 Interdisciplinary Graduate Program in Immunology, University of Iowa, Iowa City, Iowa, United States of America; 2 Department of Pathology, University of Iowa, Iowa City, Iowa, United States of America; 3 Interdisciplinary Graduate Program in Molecular Medicine, University of Iowa, Iowa City, Iowa, United States of America; 4 Department of Microbiology and Immunology, University of Iowa, Iowa City, Iowa, United States of America; 5 Gastroenterology Division, Department of Medicine, Washington University School of Medicine, St. Louis, Missouri, United States of America; 6 Department of Immunology and Microbiology, Thomas Jefferson University, Philadelphia, Pennsylvania, United States of America; 7 Microbiology, Immunology, and Cancer Biology Ph.D. Program, University of Minnesota, Minneapolis, Minnesota, United States of America; 8 Center for Immunology, University of Minnesota, Minneapolis, Minnesota, United States of America; 9 Department of Urology, University of Minnesota, Minneapolis, Minnesota, United States of America; 10 Minneapolis VA Health Care, University of Minnesota, Minneapolis, Minnesota, United States of America; University of Zurich, SWITZERLAND

## Abstract

The sepsis-induced cytokine storm leads to severe lymphopenia and reduced effector capacity of remaining/surviving cells. This results in a prolonged state of immunoparalysis, that contributes to enhanced morbidity/mortality of sepsis survivors upon secondary infection. The impact of sepsis on several lymphoid subsets has been characterized, yet its impact on NK-cells remains underappreciated–despite their critical role in controlling infection(s). Here, we observed numerical loss of NK-cells in multiple tissues after cecal-ligation-and-puncture (CLP)-induced sepsis. To elucidate the sepsis-induced lesions in surviving NK-cells, transcriptional profiles were evaluated and indicated changes consistent with impaired effector functionality. A corresponding deficit in NK-cell capacity to produce effector molecules following secondary infection and/or cytokine stimulation (IL-12,IL-18) further suggested a sepsis-induced NK-cell intrinsic impairment. To specifically probe NK-cell receptor-mediated function, the activating Ly49H receptor, that recognizes the murine cytomegalovirus (MCMV) m157 protein, served as a model receptor. Although relative expression of Ly49H receptor did not change, the number of Ly49H^+^ NK-cells in CLP hosts was reduced leading to impaired *in vivo* cytotoxicity and the capacity of NK-cells (on per-cell basis) to perform Ly49H-mediated degranulation, killing, and effector molecule production *in vitro* was also severely reduced. Mechanistically, Ly49H adaptor protein (DAP12) activation and clustering, assessed by TIRF microscopy, was compromised. This was further associated with diminished AKT phosphorylation and capacity to flux calcium following receptor stimulation. Importantly, DAP12 overexpression in NK-cells restored Ly49H/D receptors-mediated effector functions in CLP hosts. Finally, as a consequence of sepsis-dependent numerical and functional lesions in Ly49H^+^ NK-cells, host capacity to control MCMV infection was significantly impaired. Importantly, IL-2 complex (IL-2c) therapy after CLP improved numbers but not a function of NK-cells leading to enhanced immunity to MCMV challenge. Thus, the sepsis-induced immunoparalysis state includes numerical and NK-cell-intrinsic functional impairments, an instructive notion for future studies aimed in restoring NK-cell immunity in sepsis survivors.

## Author summary

Sepsis is an exaggerated host response to infection that can initially lead to significant morbidity/mortality and a long-lasting state of immunoparalysis in sepsis survivors. Sepsis-induced immunoparalysis functionally impairs numerous lymphocyte populations, including NK-cells. However, the scope and underlying mechanisms of NK-cell impairment and the consequences for NK-cell-mediated pathogen control remain underappreciated. NK-cells contribute to early host control of pathogens through a balance of activating and inhibitory receptors, and alterations in the number and capacity of NK-cells to exert receptor-mediated immunity can lead to dramatic impairment in host control of infection. The present study defines sepsis-induced numerical and cell-intrinsic functional impairments in NK-cell response to cytokine stimulation and receptor signaling that contribute to impaired host capacity to mount NK-cell-mediated effector responses and provide protection to bacterial and/or viral pathogens. Impairments in receptor signaling were due to reduced expression of adaptor protein DAP12. Importantly, the diminished ability of NK-cells from CLP hosts to provide anti-viral (MCMV) immunity is partially restored by IL-2 complex (IL-2c) therapy, which increased the number, but not function, of protective Ly49H^+^ NK-cells. Thus, these findings define sepsis-induced changes of the NK-cell compartment and provide insight into potential therapeutic interventions aimed at resolving sepsis-induced immunoparalysis in sepsis survivors.

## Introduction

Sepsis is fatal to approximately 250,000 Americans every year and presents a significant economic burden (>$20 billion annually). The cytokine storm, which characterizes a septic event, is the result of a mismanaged infection and is composed of both pro- and anti-inflammatory cytokines [1]. However, the ~75% of patients that survive the cytokine storm can enter a state of chronic immunoparalysis associated with increased susceptibility to unrelated secondary infection, increased viral reactivation, and decreased 5-year survival compared with control cohorts [2–6]. Additionally, the sepsis-induced cytokine storm is associated with apoptosis of lymphocytes leading to severe and transient lymphopenia [7–11].

While the impact of sepsis on several lymphoid populations has been explored, including work from our labs examining the effect of sepsis on CD4 and CD8 T cell responses, the influence of sepsis on the NK-cell compartment remains understudied [9, 12–20]. The predominant focus of research to date has been on the contribution of NK-cells to sepsis severity, due to NK-dependent release of cytokines during the cytokine storm [21–30]. As such, NK-cells are largely considered detrimental in the context of sepsis, however, NK-cells are also important early mediators in the control of infection. Thus, sepsis-induced impairment in NK-cell function(s) may contribute to the increased host susceptibility to unrelated infection(s). Indeed, the enhanced susceptibility of sepsis patients to secondary infection(s) and viral re-infections indicate potential long-term impairment of NK-cells after sepsis. Additionally, there is some indication that sepsis leads to apoptosis and functional impairment of NK-cells [31–38]. However, robust characterization of these lesions, the underlying mechanisms of the sepsis-induced NK-cell dysfunction, and the direct consequences of sepsis-induced NK-cell impairment to host health remain to be elucidated.

NK-cells can be activated either by cytokine stimulation and/or receptor stimulation, and receptor expression and signaling is key to NK-cell receptor-mediated function. NK-cells recognize target cells through a balance of activating and inhibitory receptors [39–42], and when the balance is shifted in favor of activating receptors an immune synapse is formed to promote NK-cell effector function (e.g. cytokine release and cytolysis of the target cell). This process is contingent on multiple signaling events including activation of signaling cascades, incorporating events such as phosphorylation of AKT and calcium flux [42–44]. Consequently, even minor changes in these pathways can lead to cumulative downstream impairment [45]. Thus, sepsis-induced qualitative changes in NK-cell receptor-mediate function, compounding with the lymphopenic state, may lead to dramatic impairment in NK-cell-mediated control of pathogens.

Herein we describe sepsis-induced numerical loss and cell-intrinsic changes in NK-cells in response to cytokine and receptor signaling, associated with functional impairment and reduced pathogen control. While sepsis led to numerical loss, it did not alter the subset composition of surviving NK-cells. However, alterations in NK-cell-mediated cytotoxicity gene transcription and impaired receptor signaling were observed following sepsis induction. These cell-intrinsic changes were associated with a per-cell functional impairment in response to both cytokine stimulation and specific receptor stimulation. As a consequence, mice had reduced NK-cell-mediated pathogen control that could be improved by therapeutic administration of interleukin-2 complexes (IL-2c). This improved pathogen control was a result of an increased number, but not inherent function, of NK-cells following IL-2c therapy.

## Results

### Apoptosis contributes to sepsis-induced systemic loss of NK-cells

**Fig 1:**
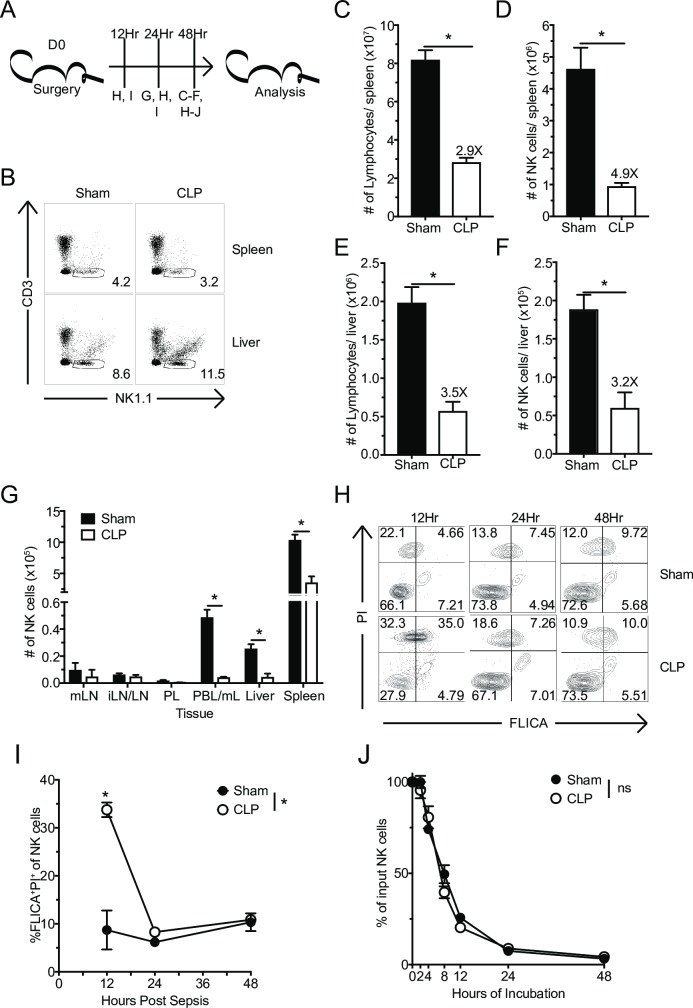
Apoptosis contributes to sepsis-induced systemic loss of NK-cells. (A) Experimental Design. Sham or CLP mice were sacrificed 12, 24, or 48 hrs after surgery, and the number of NK-cells in the indicated tissues evaluated. (B) Representative flow plots of NK-cell gating. The total number of lymphocytes or NK-cells in spleen (C,D) or liver (E,F) 48 hrs after Sham or CLP surgery. (G) The number of NK-cells in mesenteric (mLN) and inguinal lymph nodes (iLN), peritoneal lavage (PL), blood (PBL/mL), liver, and spleen 24 hrs after sepsis-induction. (H) Representative flow plots of FLICA and PI staining of NK-cells. (I) Frequency of apoptotic (FLICA^+^PI^+^) NK-cells in the spleen at 12, 24, and 48 hrs after sepsis induction. (J) NK-cells obtained from Sham and CLP host 48 hrs post-surgery were placed in *in vitro* culture and the percent of surviving NK-cells was determined at indicated times. Data are representative from 3 independent experiments with 3–5 mice per group. Numbers above bars show fold change between groups. * p<0.05. Error bars represent the standard error of the mean.

NK-cell-mediated protection against infection is contingent on the number of NK-cells capable of recognizing the presence of pathogen at the time of infection [46]. Sepsis leads to a loss of several lymphocyte populations, including NK-cells, in a variety of tissues [8, 47, 48]. To further examine the impact of sepsis on the NK-cell compartment, inbred C57Bl/6 (B6) mice underwent Sham (control) or cecal-ligation and puncture (CLP) surgery. At early stages of the sepsis-induced immunoparalysis state, day 2 post-CLP surgery (at the time when cytokine storm is diminished/absent [17, 49]), spleens and livers were harvested (**Fig 1A**). NK-cells were identified as NK1.1^+^ (activating receptor expressed early in NK-cell development) and CD3^-^, to exclude natural killer T lymphocytes (NKT) (**Fig 1B**) [50, 51]. Consistent with existing data, total lymphocyte and NK-cell numbers were dramatically diminished in both the spleen (**Fig 1C and 1D**) and liver (**Fig 1E and 1F**) from CLP-treated mice compared to Sham counterparts [8, 47]. However, no alteration in the frequency of NK-cells in either of these tissues was observed.

This decline in NK-cell numbers during the early immunoparalysis state suggests that CLP leads to apoptosis of NK-cells. Alternatively, as a result of initial sepsis-induced inflammation, NK-cells can follow inflammatory cues and be represented in substantially higher numbers at locations proximal to the initial insult. To address these possibilities NK-cell numbers were determined in proximal (e.g. mesenteric lymph node (mLN), peritoneal lavage (PL)) and/or distal sites (e.g. inguinal lymph nodes (iLN)) 24 hrs post-sepsis induction. A numerical loss of NK-cells was observed across all tissues analyzed, suggesting that redistribution, including to the abdominal cavity, does not account for overall decline in numbers (**Fig 1G**). To address whether apoptosis contributed to the loss of NK-cells, spleen-derived NK-cells were evaluated for expression of activated caspase (FLICA) and the loss of membrane integrity (propidium iodide [PI]) at 12, 24, and 48 hrs after sepsis induction. Importantly, a significant increase in the frequency of apoptotic (FLICA^+^PI^+^) NK-cells was observed in septic hosts 12 hrs after sepsis induction, which resolved by 24 and 48 hrs (**Fig 1H**) suggesting that apoptosis of NK-cells occurs early and is transient. To further explore the timing of numerical decline (apoptosis) of NK-cells after sepsis induction, splenocytes from Sham and CLP hosts at 48 hrs post-surgery were harvested and placed in culture for an additional 48 hrs (**Fig 1J**). Importantly, the number of NK-cells recovered over 48 hrs was indistinguishable between the two groups suggesting that potential ‘intrinsic’ differences in NK-cell compartment could not be attributed to increased frequency of dying NK-cells (**Fig 1H–1J**).

Multiple cell death pathways, including receptor- and mitochondria-mediated cell-death pathways, contribute to sepsis-induced apoptosis and lymphopenia [52, 53]. Yet fratricide has been shown to contribute to NK-cell loss following infection [54]. To address whether fratricide was also a factor in the NK-cell loss during sepsis, splenocytes were transferred into Thy1 disparate WT and perforin knockout (*Prf*^*-/-*^) mice (**S1A Fig**). Thy1.1^+^ (transferred) NK-cells were identified in the spleen 2 days after sepsis induction and the fold loss was calculated for both WT and *Prf*^*-/-*^ mice. We did not observe a difference in fold loss of NK-cells between WT and *Prf*^*-/-*^ mice (**S1B–S1D Fig**), suggesting fratricide did not contribute for the loss of NK-cells during sepsis.

While inbred mouse strains, such as B6, are valuable for in-depth analyses of NK-cells with well-defined activating and inhibitory receptor repertoires, the genetic homogeny within individual strains does not reflect the true genetic diversity observed in human population [55]. To compensate for this lack of genetic diversity we have previously utilized outbred Swiss Webster (SW) mice to evaluate immunologic responses in genetically heterogeneous populations [17, 19, 56, 57]. To determine the extent to which the loss of NK-cells following sepsis was recapitulated in genetically heterogeneous population, we performed Sham or CLP surgery on outbred SW mice (**S2A Fig**). Similar to data from inbred B6 mice, a decline in total NK-cell (here defined as NKp46^+^CD3^-^(**S2B Fig**)) numbers in both the spleen (**S2C Fig**) and liver (**S2D Fig**) was observed. These data suggest sepsis-induced numerical loss of NK-cells represents a global phenomenon not restricted to particular inbred strain of mice.

### Sepsis induces changes in molecular pathways relevant to NK-cell effector function

**Fig 2:**
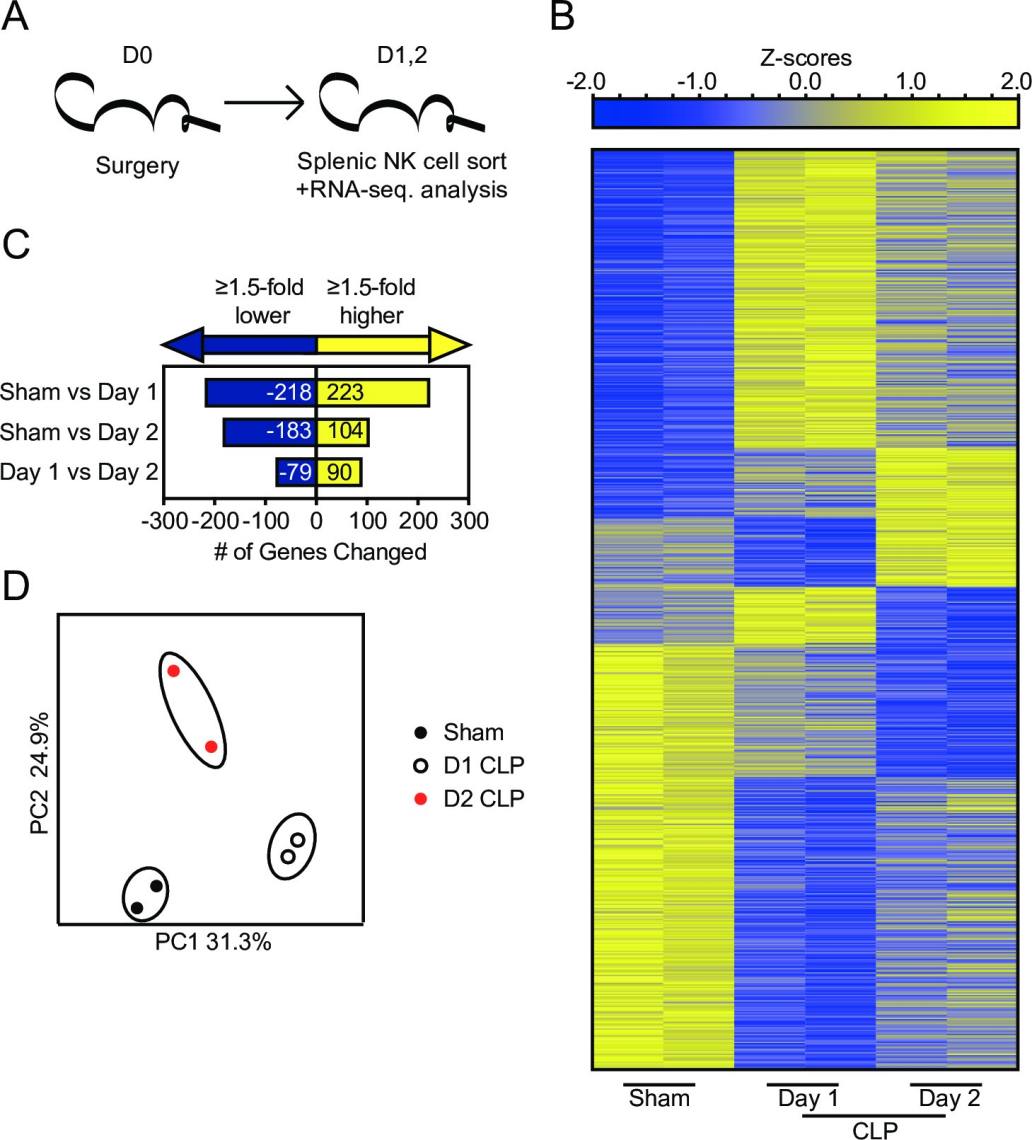
Sepsis induces significant transcriptional changes in NK-cells. (A) Experimental Design. Mice were sacrificed at day 1 or 2 post-Sham or CLP surgery and NK1.1^+^/CD3^-^ NK-cells FACS-sorted from the spleen before RNA extraction. (B) Gene expression heatmap of genes with statistically significant change (Fold change greater than or equal to 1.5 and p<0.05) as a result of any combination of comparison. (C) Number of statistically significant gene changes as a result of each comparison. (D) Principal component analysis of significantly changed genes.

**Fig 3:**
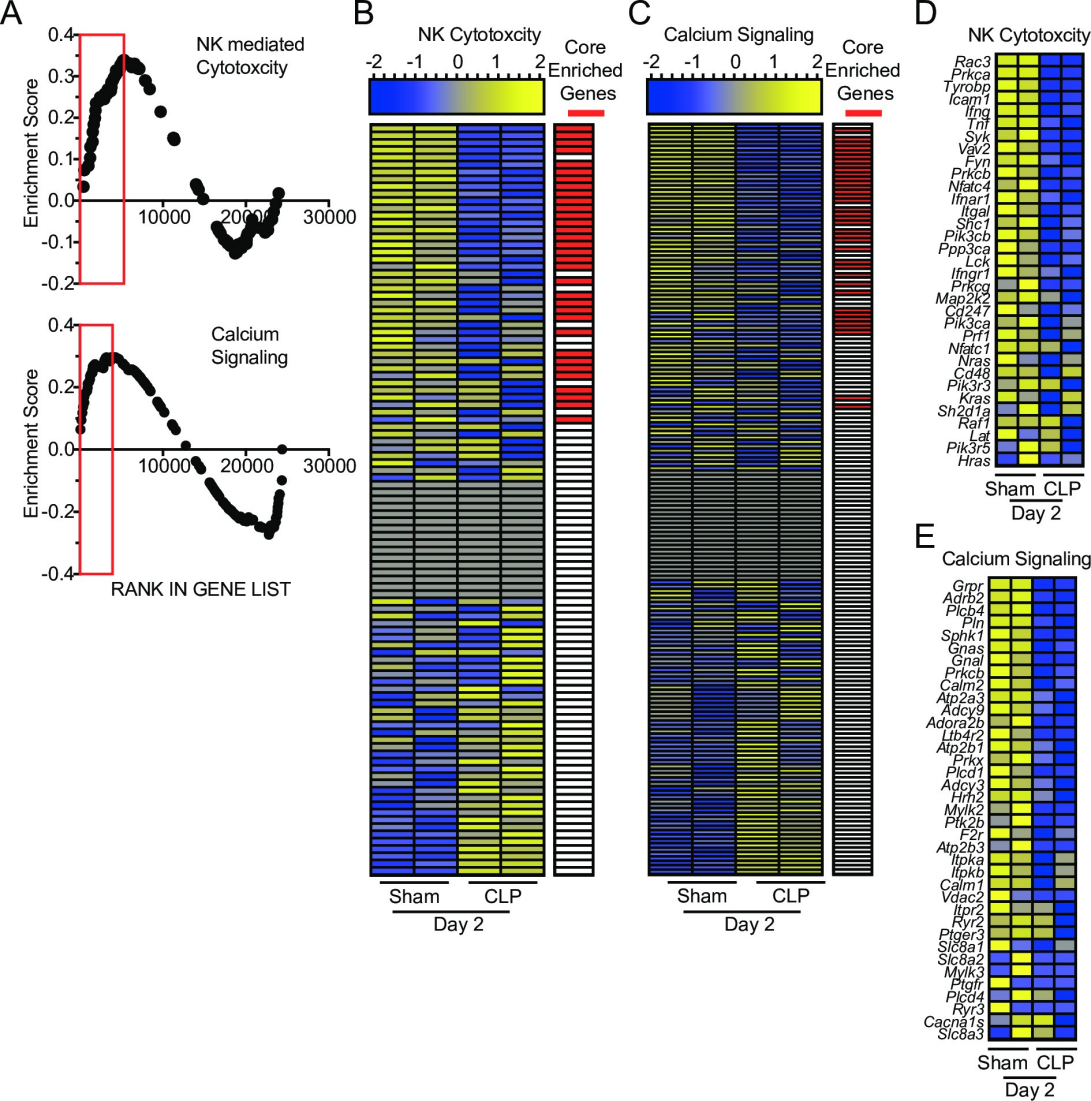
Gene-set enrichment analysis (GSEA) reveals sepsis-induced changes in molecular pathways relevant to NK-cell effector functions. (A) Enrichment scores of genes in pathways relevant to sepsis (apoptosis) and NK-cells functionality (NK-mediated cytotoxicity and calcium signaling). Red box indicates leading edge of enriched region; negative enrichment—box to left, positive enrichment—box to right. Gene expression heatmap and core enrichment of genes in NK-cell-mediated Cytotoxicity (B) and Calcium Signaling (C). Gene expression heatmap of core enriched genes in NK-cell-mediated Cytotoxicity (D) and Calcium Signaling (E).

Sepsis could lead to numerical decline of NK-cells and changes in effector capability of NK-cells [31–38]. To address how sepsis alters the functional capacity of NK-cells we evaluated the NK-cell transcriptome after sepsis (via RNA-sequencing; RNA-seq.) coupled with gene-set enrichment analysis (GSEA) (**Figs 2 and 3**). GSEA uses enrichment scores to compare the enrichment of genes in a ranked list. For this analysis RNA was isolated and sequenced from sorted splenic NK1.1^+^CD3^-^ NK-cells derived from Sham and CLP mice at days 1 or 2 post-surgery (**Fig 2A**). We detected 441 and 287 genes that are differentially expressed in NK-cells (fold change ≥ 1.5, p<0.05) between day 2 Sham and day 1 and 2 CLP NK-cells, respectively. Additionally, we noted 169 differentially expressed genes between day 1 and day 2 CLP NK-cells, demonstrating an evolving transcriptional profile as the cytokine storm develops and resolves. **Fig 2B** shows a heat map of genes with significantly different expression between NK-cells; gene changes are enumerated in **Fig 2C**. Principal component analysis of gene expression subsequently identified distinct clustering of sampled groups, indicating time-dependent changes in NK-cell gene expression (**Fig 2D**).

GSEA was performed comparing day 2 Sham and CLP samples to address functional changes that manifest at the beginning of the immunoparalysis phase (post-cytokine storm). We focused on enriched pathways that are closely associated with NK-cell effector functions. Interestingly, CLP samples demonstrated negative enrichment for genes associated with NK-cell-mediated cytotoxicity and calcium signaling, an important component of receptor signaling for target cell recognition (**Fig 3A**). This analysis revealed impairments in NK-cell receptor signaling may be associated with the impaired effector capacity of NK-cells following sepsis. Expression of genes in these pathways were evaluated to assess how sepsis alters NK-cell function. As expected we observed core enrichment of genes reduced in expression after sepsis (**Fig 3B and 3C**). We next evaluated the expression of the core enriched genes to define potential lesions. While we did not observe changes in NK-cell receptor expression among core enriched genes, many of the modulated genes were associated with receptor signaling (e.g. *Tyrobp*, *Lat*, *Plcd1*) or effector molecules (e.g. *Ifng*, *Tnf*, *Prf1*) (**Fig 3D and 3E**). Notably, expression changes observed in receptor signaling primarily encode proteins that participate in early activating receptor signaling cascades [58–60]. Thus, they are likely to impact a wide range of receptors even if expression of the receptor itself is unaltered. These changes represent a potential cell-intrinsic perturbation in receptor signaling and cumulatively suggest a potential sepsis-induced impairment in NK-cell effector functionality.

### Sepsis impairs NK-cell capacity to produce IFN-γ in response to bacterial *L*. *monocytogenes* infection or cytokine stimulation

**Fig 4:**
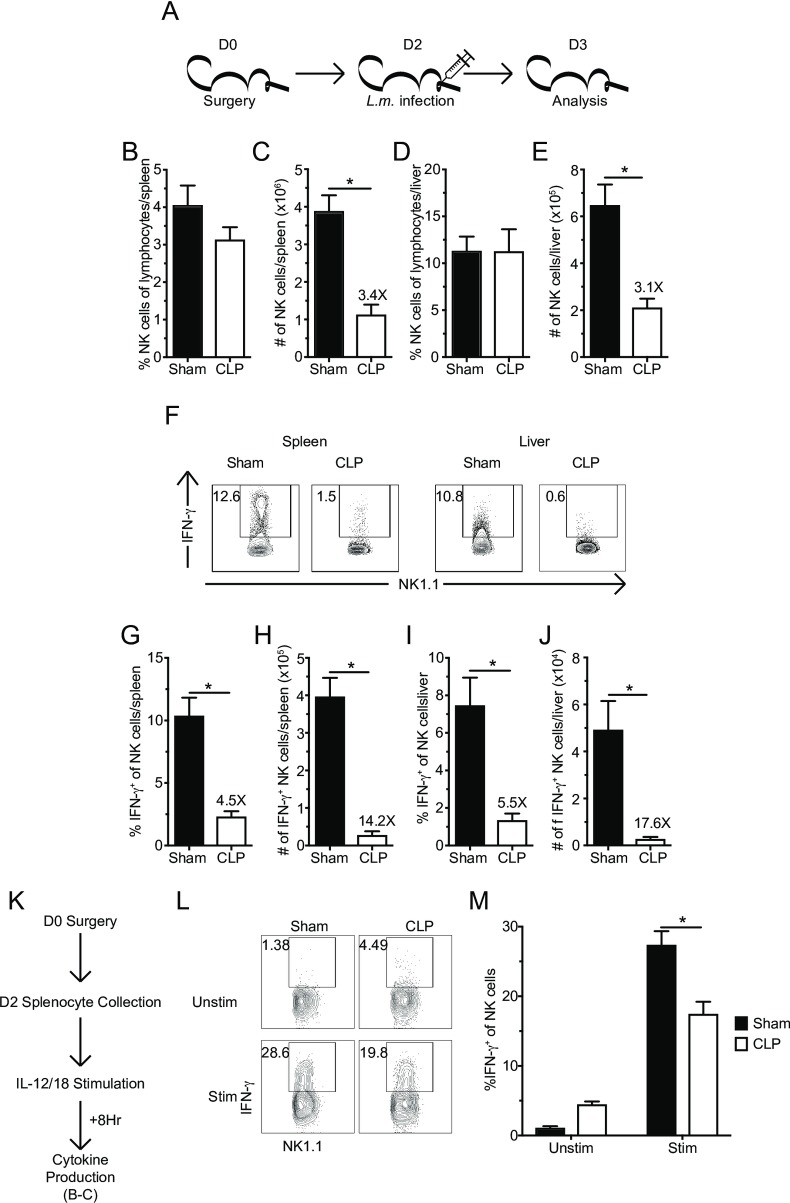
Sepsis impairs NK-cell capacity to produce IFN-γ in response to *L*. *monocytogenes* infection and cytokine stimulation. (A) Experimental Design. 2 days after sham or CLP surgery mice were infected with of virulent *Listeria monocytogenes* (*L*.*m*. - 10^4^ CFU, i.v.). The frequency or number of NK-cells in the spleen (B,C) or liver (D,E) 1 day after *L*.*m*. infection. (F) Representative flow plots of IFN-**γ** producing NK-cells. The frequency or number of IFN-**γ**^+^ NK-cells in the spleen (G,H) or liver (I,J). (K) Experimental Design: 2 days after surgery splenocytes from both groups of mice were harvested and stimulated with rIL-12 and rIL-18 or left unstimulated for 8 hrs. BFA was added during the last 4 hrs and intracellular cytokine production was evaluated. (L) Representative flow plots of IFN-**γ** producing NK-cells. (M) The frequency of IFN-**γ**^+^ NK-cells from Sham or CLP hosts in either unstimulated or stimulated wells. Data are representative from 2 independent experiments with 3–5 mice per group. Numbers above bars show fold change between groups. * p<0.05. Error bars represent the standard error of the mean.

To assess to what extent NK-cells (analyzed on the population level) in the post-sepsis environment exhibit functional impairment, as suggested by the GSEA, mice were infected with virulent *Listeria monocytogenes* (*L*.*m*.) 2 days post-sepsis induction. While *L*.*m*. is not an infection commonly associated with sepsis clinically, it can be used to directly probe NK-cell capacity to produce the effector cytokine IFN-**γ** [61, 62]. Similar to data shown before, total numbers of NK-cells were diminished in spleens and livers of CLP hosts one day after *L*.*m*. infection (**Fig 4A–4E**). Importantly, the frequency of IFN-**γ** producing NK-cells directly *ex vivo* in response to secondary infection was also reduced in both tissues of CLP hosts (**Fig 4F**). The reduced number of NK-cells and the reduced frequency of IFN-**γ** producing NK-cells results in a dramatic reduction (14 and 17-fold in the spleen and liver, respectively) in the number of IFN-**γ** producing NK-cells (**Fig 4G–4J**). Thus, these data are consistent with GSEA analysis suggesting sepsis has the capacity to influence effector functionality of the remaining NK-cells in response to secondary bacterial infection.

However, sepsis induces perturbation in the dendritic cells (DCs) and other cellular compartments could also influence pathogen-induced cytokine secretion *in vivo* (ex. DC derived IL-12 [19]) necessary to facilitate IFN-**γ** production by NK-cells. To determine the extent to which intrinsic defects contributed to the impaired production of IFN-**γ** by NK-cells during *L*.*m*. infection, NK-cells obtained from Sham and CLP hosts 48 hours after surgery were stimulated with IL-12 and IL-18 directly *ex vivo* to bypass sepsis-induced changes in endogenous levels of stimulatory cytokines [19] (**Fig 4K**). Importantly, NK-cells from CLP hosts exhibited impairment in IFN-**γ** production following cytokine stimulation compared to Sham counterparts (**Fig 4L and 4M**), which corresponded with the reduced expression of the *Il12rb1* gene identified in the RNA-seq (1.3-fold reduction in expression; p<0.05). Therefore, the data in Fig 4 collectively show sepsis reduces capacity of NK-cell compartment to produce effector cytokines in response to infection *in vivo* and/or cytokine stimulation directly *ex vivo*.

### Sepsis impairs Ly49H-mediated target cell lysis

To precisely define the extent to which sepsis affects NK-cell functional capacity we switched to a model system in which we could probe a single NK-cell receptor for its capacity to mediate effector functions and protect against infection. Ly49H, an activating receptor expressed exclusively by subpopulations of NK-cells, has no endogenous ligand and recognizes the m157 immunoevasion protein of murine cytomegalovirus (MCMV) [63, 64]. Additionally, Ly49H^+^ NK-cells are critical in the control of MCMV and expression of the receptor confers resistance to MCMV by mouse strains (such as B6) [65–67]. Thus, high ligand specificity and importance in host immunity to infection make Ly49H^+^ NK-cells an ideal population to mechanistically examine the impact of sepsis on NK-cell receptor-mediated immunity.

**Fig 5:**
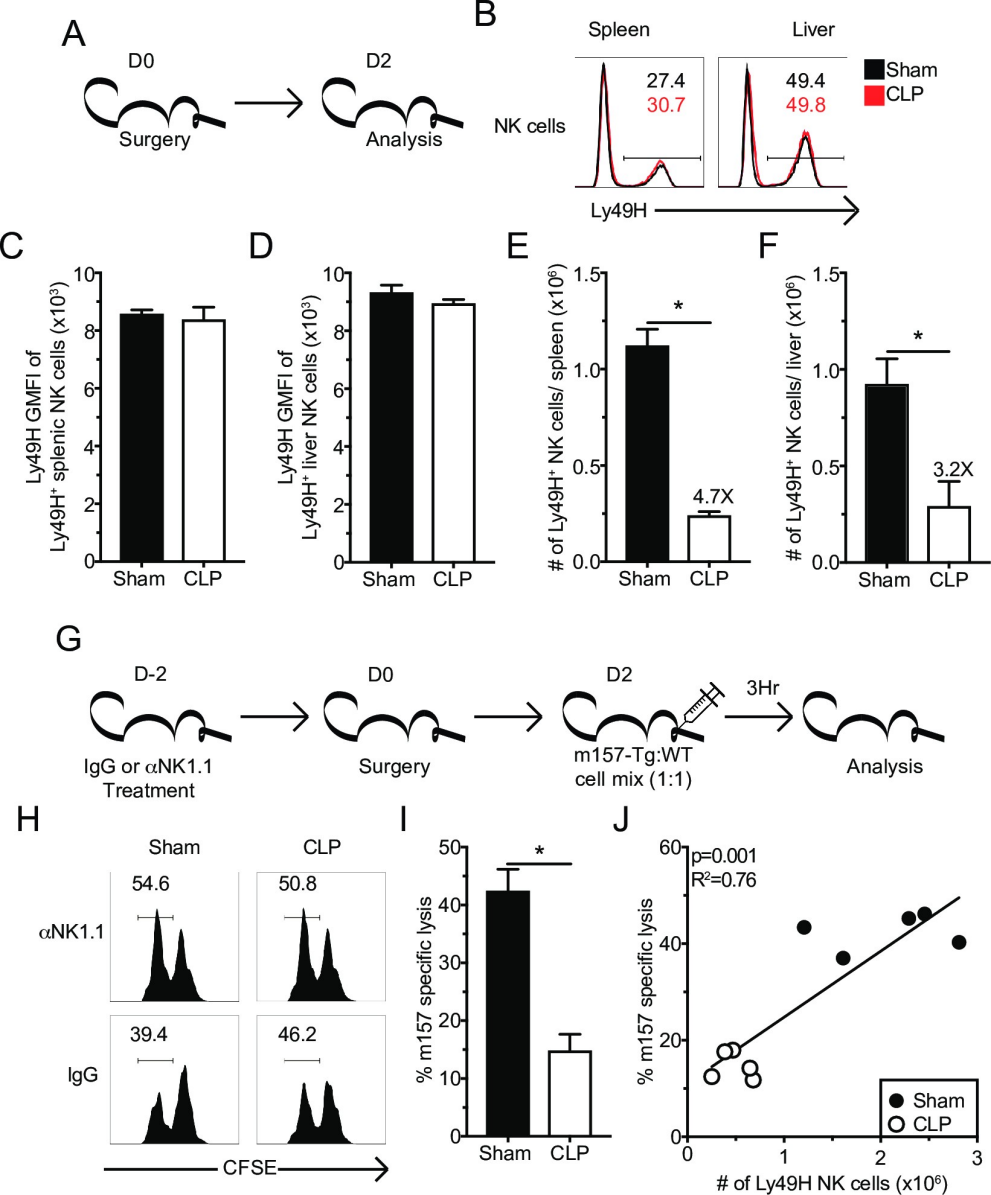
Sepsis results in numerical loss of Ly49H^+^ NK-cells and impaired *in vivo* killing. (A) Experimental Design. Mice were sacrificed 2 days after sham or CLP surgery, and the number and Ly49H expression of Ly49H^+^ NK-cells in the spleen and liver determined. (B) Representative flow plots. Numbers indicate the frequency of Ly49^+^ NK-cells. The total number or GMFI of Ly49H by Ly49H^+^ NK-cells in spleen (C,E) or liver (D,F). (G) Experimental Design. Mice were treated with control IgG or α-NK1.1 depleting antibody prior to sepsis induction. Two days post-surgery all groups of mice received a 1:1 mixture of CFSE-labeled m157 expressing (m157-Tg) target (CFSE^lo^) and m157-deficient littermate (WT) control (CFSE^hi^) cells. 3 hrs after injection mice were sacrificed and the ratio of m157-Tg to WT cells was determined. NK-depleted mice served as controls. (H) Representative flow plots. (I) m157 specific lysis in the spleen after Sham or CLP. (J) Correlation of m157 specific lysis with the number of Ly49H^+^ NK-cells in the spleen. Data are representative from 3 independent experiments with 3–5 mice per group. Numbers above bars show fold change between groups. * p<0.05. Error bars represent the standard error of the mean.

To determine the extent to which sepsis affects the frequency Ly49H^+^ NK-cells and the level of Ly49H expression, NK-cells from the spleens and livers were evaluated 2 days after surgery (**Fig 5A**). Although sepsis did not change the frequency and/or relative expression of Ly49H on NK-cells (**Fig 5B–5D**), numerical loss of the Ly49H^+^ NK-cells was observed in both the spleens (**Fig 5E**) and livers (**Fig 5F**) suggesting similar susceptibility of Ly49H^+^ NK-cells to sepsis-induced apoptosis. Additionally, the maturation status of NK-cells determines their function [68, 69]. To address whether sepsis altered the maturation of surviving NK-cells, subset composition of Ly49H^+^ NK-cells was determined by CD27 and CD11b expression [68]. Additionally, KLRG1 and Ly6C were used as markers of NK-cell terminal maturation [70–72]. As predicted, numerical loss of all subsets was observed in the spleen and liver; however, no changes in the composition of NK subpopulations was detected (**S3A–S3G Fig**). Finally, inflammation can alter NK-cell expression of effector molecules [73]. Thus, we sought to address the extent to which sepsis altered the steady state expression of Granzyme B (GzmB) in NK-cells. Baseline GzmB expression of NK-cells was not impacted by sepsis (**S3H–S3J Fig**). Therefore, sepsis induces the loss of Ly49H^+^ NK-cells without changing the maturation status and/or levels of Ly49H receptor expression on NK-cells.

To define if sepsis influences the killing capacity of Ly49H^+^ NK-cells, wild type (WT) and m157-expressing splenocytes (m157-Tg) were used as targets in an *in vivo* cytotoxic assay [66, 74]. Two days post-sepsis induction target cells, CFSE-labelled m157-Tg (CFSE^lo^) and WT (CFSE^hi^) splenocytes were injected in a 1:1 mixture i.v. into Sham- and CLP-treated mice. Additional groups of mice, in which the NK-cell compartment (αNK1.1 groups) was depleted prior to the sepsis induction, were included as necessary controls (**Fig 5G**). Results in **Fig 5H and 5I** clearly indicate impaired specific lysis of m157 target cells in the spleens of CLP hosts compared to Sham controls. Importantly, the specific lysis of m157 target cells strongly correlated with the number of Ly49H^+^ NK-cells present in the spleen (**Fig 5J**). Thus, these data suggest the sepsis-imposed numerical loss of Ly49H^+^ NK-cells resulted in impaired target cell killing *in vivo*.

### Sepsis-induces NK-cell-intrinsic functional impairments of Ly49H and Ly49D receptors

**Fig 6:**
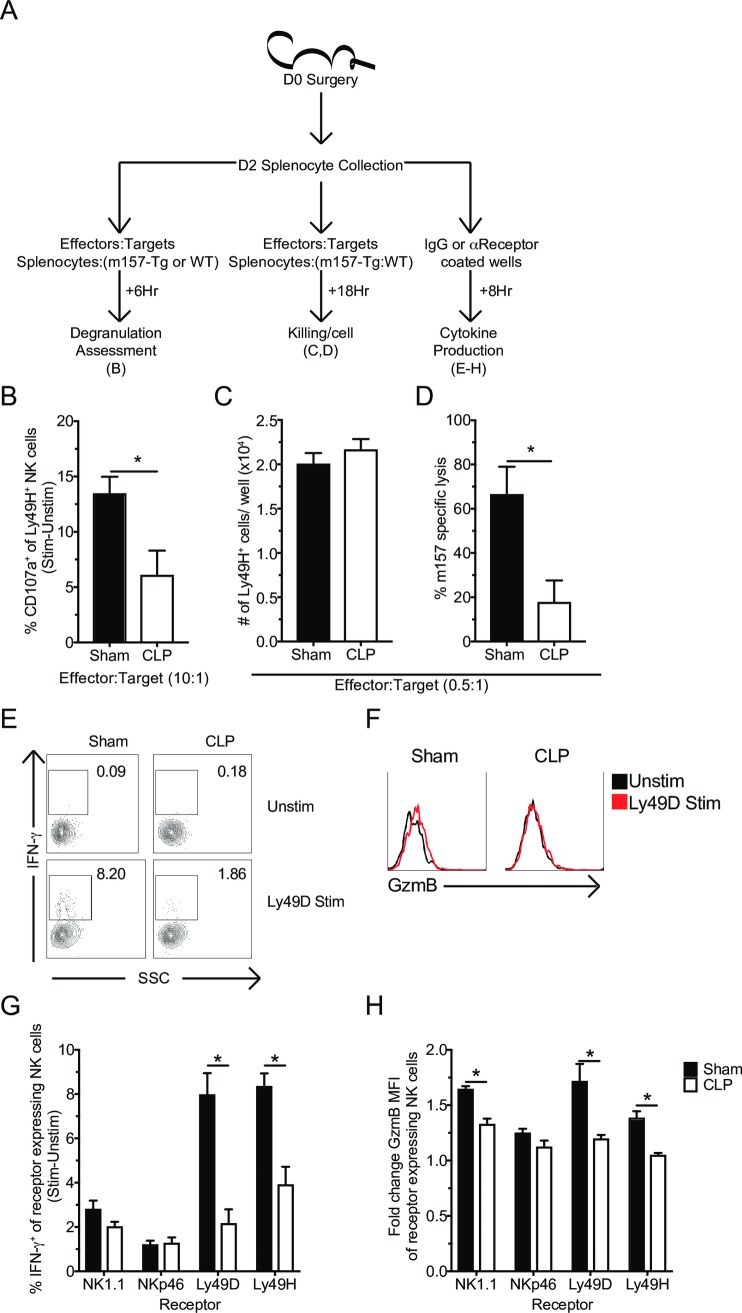
Sepsis-induces NK-cell-intrinsic functional impairments of Ly49H and Ly49D receptors. (A) Experimental Design. Splenocytes (day 2 post-surgery) were either incubated with: m157-Tg or WT cells for 6 hrs in the presence of monensin to determine ligand-induced degranulation; with m157 and WT cells for 18 hrs to determine *in vitro* killing; or 8 hrs stimulation with plate-bound antibody and BFA to determine IFN-**γ** production. (B) Frequency of CD107a^+^ Ly49H^+^ NK-cells in response to ligand (Stim[m157 targets]-Unstim[WT targets]) at indicated effector to target ratios. (C) Number of Ly49H^+^ NK-cells per well. (D) NK-cell-mediated m157 specific lysis after 18 hrs *in vitro* incubation. Representative flow plots of IFN-**γ** (E) or GzmB (F) following stimulation. Frequency of IFN-**γ**^+^ (G) or fold GzmB GMFI (H) of stimulated receptor^+^ NK-cells from Sham or CLP mice after 8 hrs stimulation with plate bound control (IgG), αNK1.1, αNKp46, αLy49D, or αLy49H antibody. Data are representative from 2 independent experiments with 3–5 mice per group. Numbers above bars show fold change between groups. * p<0.05, # p<0.05 group comparison by two-way ANOVA. Error bars represent the standard error of the mean.

While the reduced number of Ly49H^+^ NK-cells resulted in a deficit in CLP host capacity to kill target cells, the RNA-seq. data and reduced IFN-**γ** production upon cytokine stimulation also indicated NK-cells may exhibit sepsis-induced functional impairments on a per-cell basis. To assess this possibility, splenocytes from Sham and CLP mice were incubated **I)** *in vitro* with either m157-Tg or WT cells separately to assess degranulation, **II)** with the target cell mix of m157-Tg and WT splenocytes described above to assess killing, or **III)** plate bound stimulatory antibodies (or control IgG) to assess effector molecule production (**Fig 6A**). Of note: since sepsis alters the number but not the frequency of both total and Ly49H^+^ NK-cells, the capacity of Ly49H expressing NK-cells to perform effector function(s) (e.g. degranulation, target cell lysis and cytokine production) on per-cell basis was determined by plating equivalent numbers of either Sham or CLP splenocytes per well.

Relative to Sham, Ly49H^+^ NK-cells from CLP hosts exhibited less degranulation (as determined by analyzing CD107a expression) [66, 75] in response to Ly49H stimulation after 6 hrs incubation *in vitro* (**Fig 6B**). Impaired degranulation can subsequently impair the capability to kill target cells [75]. To determine the extent to which sepsis impairs the intrinsic capacity of Ly49H^+^ NK-cells to perform cytolysis splenocytes from Sham or CLP hosts were incubated with the m157-Tg: WT target mix (effector to target ratio 0.5: 1) for 18 hrs, after which m157 specific lysis was assessed. While the number of Ly49H^+^ NK-cells per well was equivalent (**Fig 6C**) there was significantly less specific lysis of m157 target cells by CLP splenocytes compared to Sham splenocytes (**Fig 6D**). Target cells were separated for the degranulation assay to avoid ‘cold target’ competition; however, the killing assay required the presence of both target cell populations in the same well. Finally, to examine if sepsis modulated the ability of subsets of NK-cells to produce effector cytokines (e.g. IFN-**γ**), Sham or CLP-derived splenocytes were stimulated with plate bound stimulatory antibodies to different activating NK-cell receptors (NK1.1, NKp46, Ly49D, or Ly49H) or control IgG antibodies [73], and we assessed IFN-**γ** production and upregulation of GzmB for cells expressing the receptor stimulated with its agonistic/specific Ab compared to IgG control. Importantly, a defect in the capacity to produce IFN-**γ** and/or upregulate GzmB in response to receptor stimulation was most prominent in Ly49H and Ly49D expressing NK-cells (**Fig 6E–6H**). This suggests that sepsis-induced receptor impairment is potentially a result of a defect(s) shared by Ly49H and Ly49D but not NK1.1 and NKp46, a notion that will be explored further. In summary, these data collectively suggest that sepsis changes the per-cell capacity of NK-cells to respond to precisely defined viral ligands or specific receptor stimulation.

### Sepsis-induced NK-cell intrinsic functional impairment is associated with reduced DAP12 adaptor protein expression and clustering

**Fig 7:**
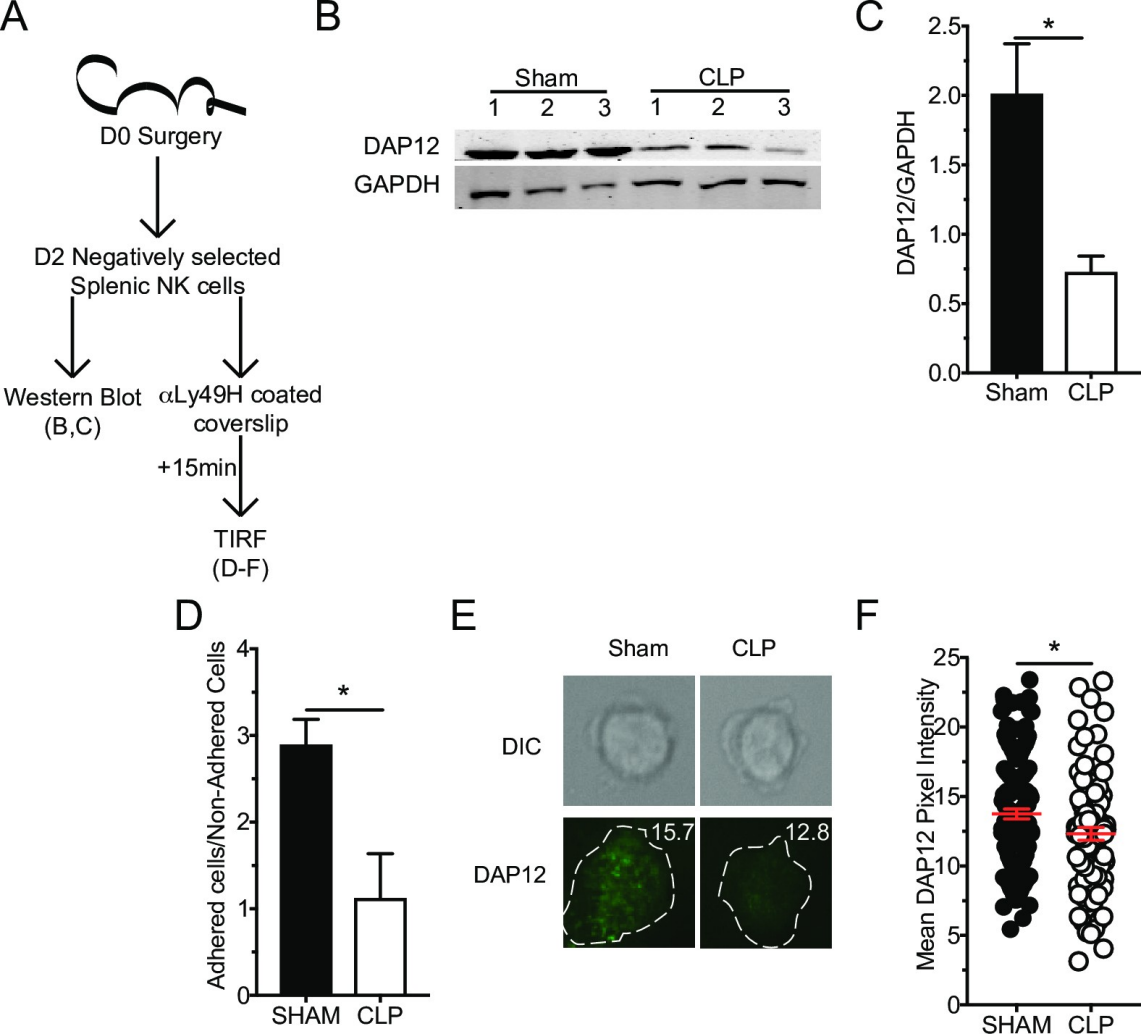
Sepsis-induced NK-cell intrinsic functional impairment is associated with reduced DAP12 adaptor protein expression and clustering. (A) Experimental Design. Splenic NK-cells were purified by negative selection 2 days post-surgery and stimulated as indicated. Immunoblot (B) and ratio quantification (C) for DAP12 and GAPDH in unstimulated NK-cells from Sham and CLP mice, numbers indicate replicates. (D) Ratio of cells obtained from Sham or CLP mice adhering to the glass slide versus non-adherent cells. (E) Representative images of DAP12 staining for TIRF microscopy. (F) Mean DAP12 pixel intensity of adhered cells. Each data set is representative from 2 independent experiments with 5x10^5^ cells per lane in panels B,C; 3 mice per group in panel D; >80 cells were analyzed per group from 3 mice per group in panels E,F. * p<0.05. Error bars represent the standard error of the mean.

Although sepsis did not alter Ly49H expression, the diminished capacity of Ly49H NK-cells to exert effector functions upon receptor ligation suggests potential lesions in receptor signaling. One of the genes indicated by RNA-seq. analysis as having reduced expression after sepsis was *Tyrobp*. This gene encodes DAP12, the adaptor protein for Ly49H (and Ly49D but not NKp46 or NK1.1) and is required for Ly49H expression and signaling [60, 66, 76, 77]. We thus hypothesized that reduced DAP12 expression contributed to impaired Ly49H signaling and subsequently impairing effector function on a per-cell basis. To address whether DAP12 expression was impacted by sepsis, NK-cells were enriched by negative selection (~85% NK-cell purity, of which ~70% were Ly49H^+^) to minimize signaling events that might occur as a result of antibody-receptor interaction. The sorted cells were then lysed and assessed for expression of DAP12 by immunoblotting using monoclonal αDAP12 antibody (**Fig 7A**). Dramatic reduction in the expression of DAP12 by NK-cells from CLP hosts was observed (**Fig 7B and 7C**). Thus, sepsis impairs expression of Ly49H/D adaptor protein DAP12 in NK-cells.

Because of the proximity of the lesion to the receptor it is likely the clustering of the receptor with its adaptor protein necessary for proper immune synapse formation may be impaired [78]. To address this possibility, NK-cells were again negatively sorted before adherence to an α-Ly49H mAb coated slide. Cells were allowed to interact with the antibody for 15 minutes before being fixed and stained with αDAP12. Slides were then examined by total internal reflection fluorescence (TIRF) microscopy, which allows for imaging of the plasma membrane at the interface with the glass slide. This allows evaluation of microcluster formation, as assessed by DAP12 density, at the site of receptor activation (**Fig 7D–7F**). Of important note, enriched NK-cells obtained after CLP surgery showed a statistically significant impairment in their ability to adhere to the slide. This is a receptor-mediated event, thus non-adherent cells that are potentially the most influenced by sepsis are, by necessity, excluded from TIRF analysis (**Fig 7D**). To further corroborate this, the reduced capacity of NK-cells obtained from CLP hosts to adhere was observed in response to various concentrations of αLy49H Abs (**S4A and S4B Fig**). Thus, the reduced expression of DAP12, coupled with reduced microcluster formation, contributes to the diminished Ly49H-mediated effector responses.

### Reduced DAP12 expression is associated with defects in receptor signaling events

Impaired DAP12 expression should also alter subsequent signaling events, as even relatively minor changes in upstream signaling can lead to dramatic differences in functional outcome [45]. While there are many signaling events that follow Ly49H receptor stimulation, AKT phosphorylation and calcium flux in the NK-cells from CLP hosts were further examined because of their relative proximity to receptor stimulation and the negative enrichment of calcium signaling associated genes identified in the RNA-seq. analysis [79].

**Fig 8:**
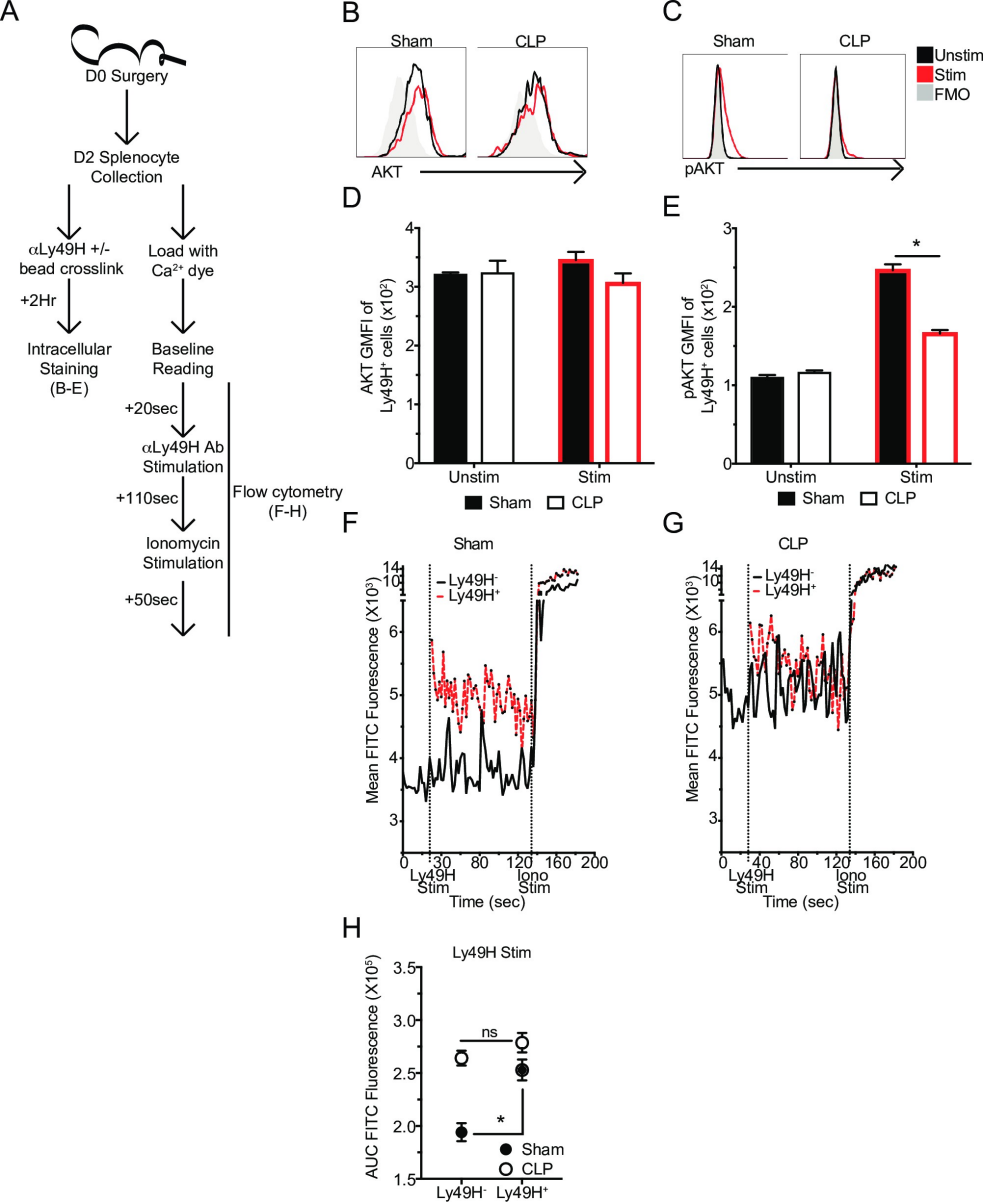
Reduced DAP12 expression is associated with defects in receptor signaling events. (A) Experimental Design. Splenocytes were obtained 2 days post-surgery and were either labeled with α-Ly49H mAb and crosslinked with beads for 2 hrs and stained with AKT and pAKT antibodies or labeled with a calcium dye before being analyzed for calcium flux (baseline– 20 sec., Ly49H stim– 110 sec., Ionomycin– 50 sec.). Representative profiles or GMFI of AKT (B,D) or pAKT (C,E) in unstimulated and stimulated Ly49H^+^ NK-cells. Mean calcium dye fluorescence of Sham (F) or CLP (G) Ly49H^+^ or Ly49H^-^ NK-cells during stimulation time course. (H) Area under the curve (AUC) for Ly49H^-^ and Ly49H^+^ NK-cells during stimulation with α-Ly49H mAb. Data are representative from 2 independent experiments with 3 mice per group. * p<0.05. Error bars represent the standard error of the mean.

To determine how sepsis alters Ly49H receptor capacity to induce AKT phosphorylation splenocytes were harvested and labeled with α-Ly49H mAb. Addition of cross-linking beads was used to stimulate the cells for 2 hrs, while no bead addition was used for unstimulated controls (**Fig 8A**). To ensure the results were not affected by a sepsis-induced change in total AKT, the amount of AKT present in stimulated and unstimulated Sham and CLP samples was evaluated and determined to be equivalent (**Fig 8B and 8D**). Unstimulated Sham and CLP cells did not have detectable pAKT(pS473, activating phosphorylation of AKT [79]), compared to FMO (fluorescence-minus-one) controls. However, upon stimulation pAKT could be detected in Sham cells but not in CLP cells (**Fig 8C and 8E**). These data indicate that while sepsis does not alter AKT expression it does alter the capacity of Ly49H to induce activating phosphorylation of AKT following receptor stimulation.

To evaluate how sepsis affects calcium signaling in NK-cells following specific receptor stimulation, splenocytes were harvested and stained to identify NK-cells (NK1.1^+^CD3^-^) before being labeled with a calcium sensitive dye (Fluo-4-AM) to detect free intracellular calcium (fluorescence correlates with calcium concentration) (**Fig 8A**) [80]. A baseline reading was taken to determine calcium concentration in the absence of stimulation. Intriguingly, NK-cells from CLP hosts had higher internal calcium at baseline indicating a potential dysregulation in calcium sequestration (**Fig 8F and 8G**). It is important to note that in the context of calcium signaling during receptor stimulation, the most important factor is how much calcium fluxes in response to receptor stimulation rather than the total amount of calcium present in the cell [77, 80]. Therefore, baseline readings were followed by stimulation with the addition of a fluorescently labeled α-Ly49H mAb. This had the additional benefit of distinguishing stimulated (Ly49H^+^) from unstimulated (Ly49H^-^) NK-cells for an effective internal control. We found that while Sham Ly49H^+^ NK-cells fluxed calcium efficiently (relative to Ly49H^-^ NK-cells), CLP Ly49H^+^ NK-cells did not (**Fig 8F and 8G**). This was further quantified using area under the curve (AUC) of Ly49H^+^ or Ly49H^-^ NK-cell populations as a surrogate measurement of internal calcium flux during the stimulation period (**Fig 8H**). Finally, results were validated by stimulating with Ionomycin (Iono) to determine that the peak amount of calcium present in all cells was similar suggesting that differences were the result of different capacity to flux calcium rather than different total amounts of calcium (**Fig 8F and 8G**). Thus, these results suggest sepsis impairs the ability of NK-cells to respond to viral ligands due to reduced expression of the receptor’s adaptor protein leading to impairments in downstream signaling, including fluxing calcium and phosphorylating AKT, and reducing formation of the immunological synapse.

### Sepsis-induced loss of DAP12 is causal in NK-cell-intrinsic impairments

**Fig 9:**
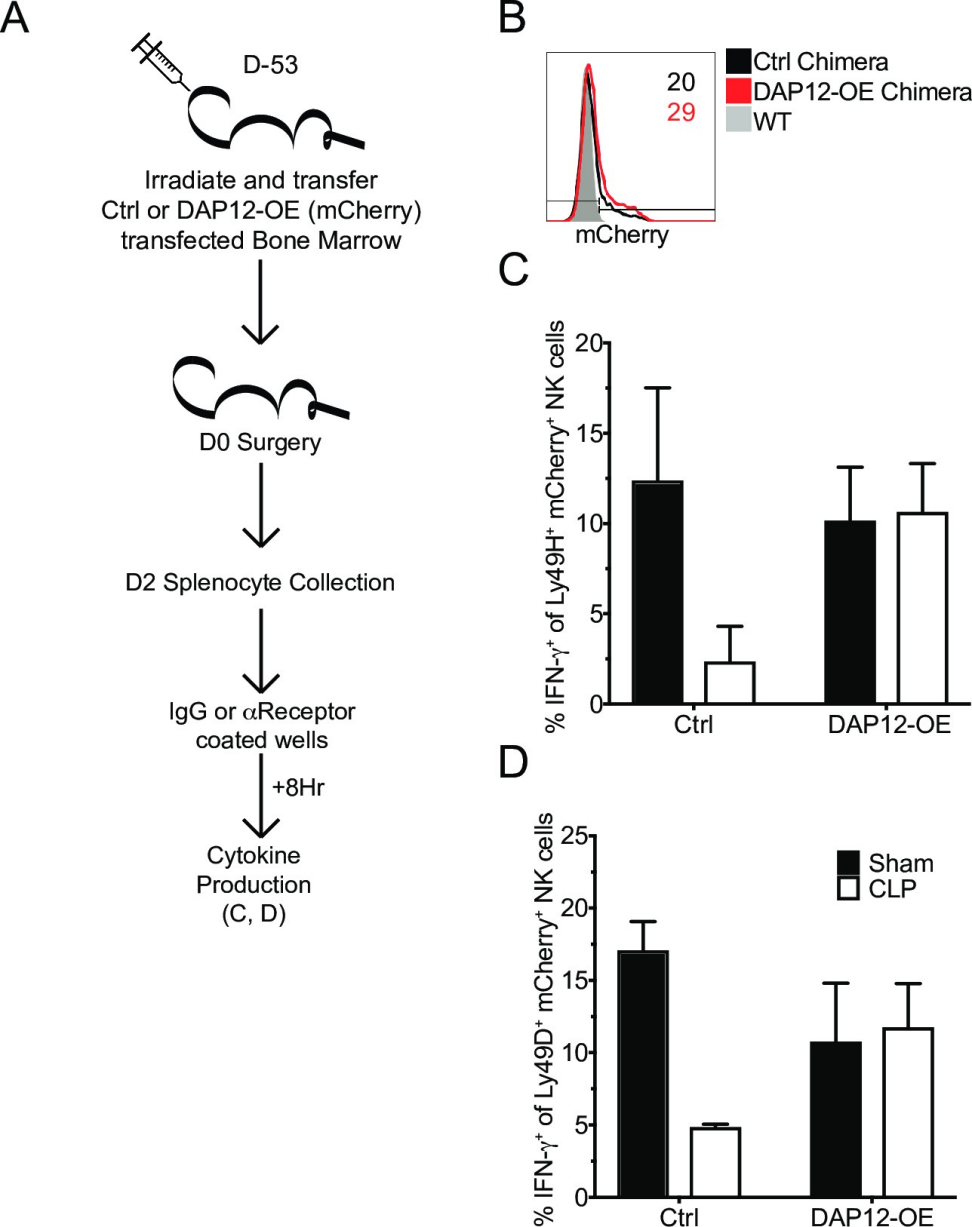
Sepsis-induced loss of DAP12 is causal in NK-cell-intrinsic impairments. (A) Experimental Design. BM chimeras containing mCherry^+^ control (Ctrl) vector or DAP12-overexpression (DAP12-OE) were generated. Following reconstitution mice underwent Sham or CLP surgery. 2 days after surgery IFN-**γ** production in response to DAP12-dependent receptor (Ly49H and Ly49D) stimulation was assessed. (B) Representative flow plot of mCherry expression on WT, Ctrl, and DAP12-OE Ly49H^+^ splenic NK-cells, numbers indicate frequency of mCherry+ cells. Frequency of IFN-**γ**^+^ of stimulated receptor^+^ NK-cells from Sham or CLP mice after 8 hrs stimulation with plate bound control (IgG), αLy49H (C), or αLy49D (D) antibody. Data are representative from 2 independent experiments with 2–3 mice per group. Numbers above bars show fold change between groups. * p<0.05. Error bars represent the standard error of the mean.

The previous results indicate that a loss of DAP12 is associated with the reduced functionality of Ly49H and Ly49D receptors. Therefore, to establish whether DAP12 loss is causal in impaired function of these receptors bone marrow chimeras (BM) were generated for control and DAP12 overexpression (DAP12-OE) vectors. Vectors were transfected into LSKs used for BM reconstitution and transfection was demarcated by expression of mCherry (**Fig 9A and 9B**). We next sought to address whether this rescued NK-cell DAP12-dependent receptor-mediated IFN-**γ** production of CLP hosts. Similar to previous results we observed reduced functionality of both Ly49H and Ly49D in cells from CLP Control hosts, relative to Sham counterparts. However, functionality was completely rescued by DAP12-OE in CLP hosts (**Fig 9C and 9D**). Thus, sepsis-induced loss of DAP12 expression is causal in impairment in DAP12-dependent NK-cell-receptor functionality.

The results thus far indicate proximal impairments in receptor signaling. To address whether downstream signaling impairments also exist, splenocytes were stimulated with phorbyl 12-myristate 13-acetate (PMA) and Iono to mimic DAG and calcium signaling (**S5A Fig**). PMA/Iono stimulation represents a very potent stimulus that bypasses receptor signaling. Yet even with this strong stimulation condition, both total (**S5B and S5C Fig**) and Ly49H^+^ (**S5B and S5D Fig**) NK-cells from CLP-treated mice demonstrated a significant reduction in IFN-**γ** production. These data suggest that in addition to the proximal impairment in DAP12 signaling, distal signaling events may also be impacted.

### Sepsis impairs NK-cell-mediated MCM viral control

**Fig 10:**
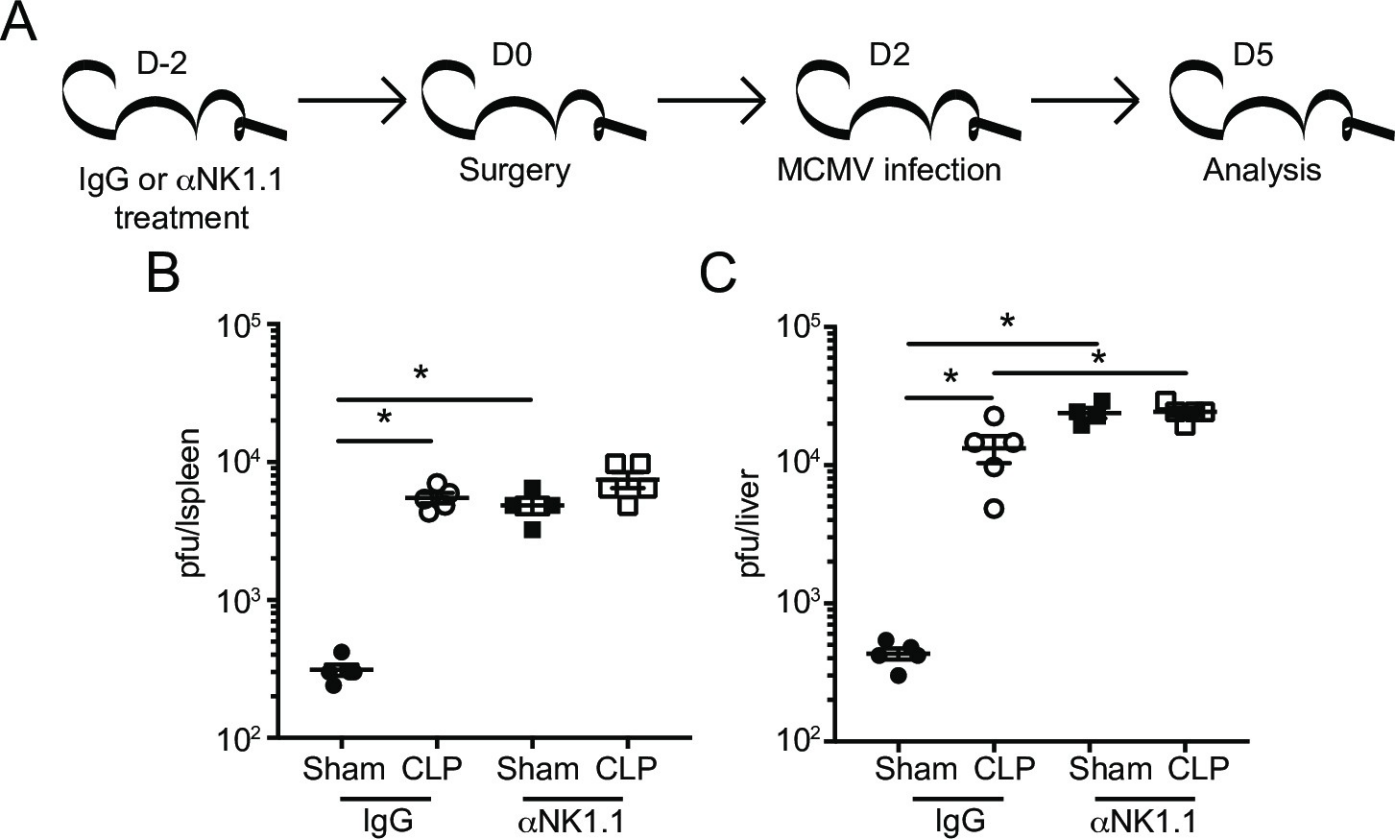
Sepsis impairs NK-cell-mediated MCM viral control. (A) Experimental Design. NK-sufficient or–deficient (IgG and α-NK1.1-treated, respectively) groups of mice were infected with the Smith strain of MCMV (10^5^ PFU, i.p.) 2 days post-Sham or CLP surgery. Viral titers in the spleens (B) or livers (C) of Sham and CLP groups 3 days after MCMV infection. (Data are representative from 3 independent experiments with 3–5 mice per group. * p<0.05. Error bars represent the standard error of the mean.

The numerical and cell-intrinsic impairments described thus far suggest pathogen control by NK-cells would be severely impaired in CLP hosts. To address this directly, MCMV was chosen as a model pathogen since Ly49H^+^ NK-cells are critical for controlling MCMV infection in B6 mice [65–67]. To test the role of NK-cells in control of MCMV in the post-sepsis environment, mice were treated with control IgG or α-NK1.1 mAb to deplete the NK-cell compartment 2 days prior to Sham or CLP surgery. Mice were subsequently infected with the Smith strain of MCMV (10^5^ PFU i.p.) 2 days after surgery. Viral titers in the spleens and livers were analyzed 3 days after infection (5 days post-surgery; **Fig 10A**) [81]. Consistent with existing data, the contribution of NK-cells is critical in providing anti-MCMV immunity since NK-depleted control (Sham) groups of mice had 15–50 fold higher viral loads in spleens and livers compared to their IgG-treated counterparts (**Fig 10B and 10C**) [66]. As expected, sepsis diminished the ability of the host to respond to MCMV challenge, and NK sufficient (IgG-treated) CLP-treated mice had a significant increase in viral load in both organs examined compared to Sham controls. Importantly, the contribution of NK-cells in anti-viral immunity *in vivo* was significantly diminished in the post-sepsis environment since similar levels of infection were observed in NK deficient (α-NK1.1 mAb group) or sufficient (IgG group) CLP hosts (**Fig 10B and 10C**). These data highlight the requirement for NK-cells in controlling MCMV infection and pinpoint the dramatic effect sepsis has on the ability of NK-cells to exert their effector functions *in vivo*.

### IL-2c therapy increases the number of Ly49H^+^ NK-cells

**Fig 11:**
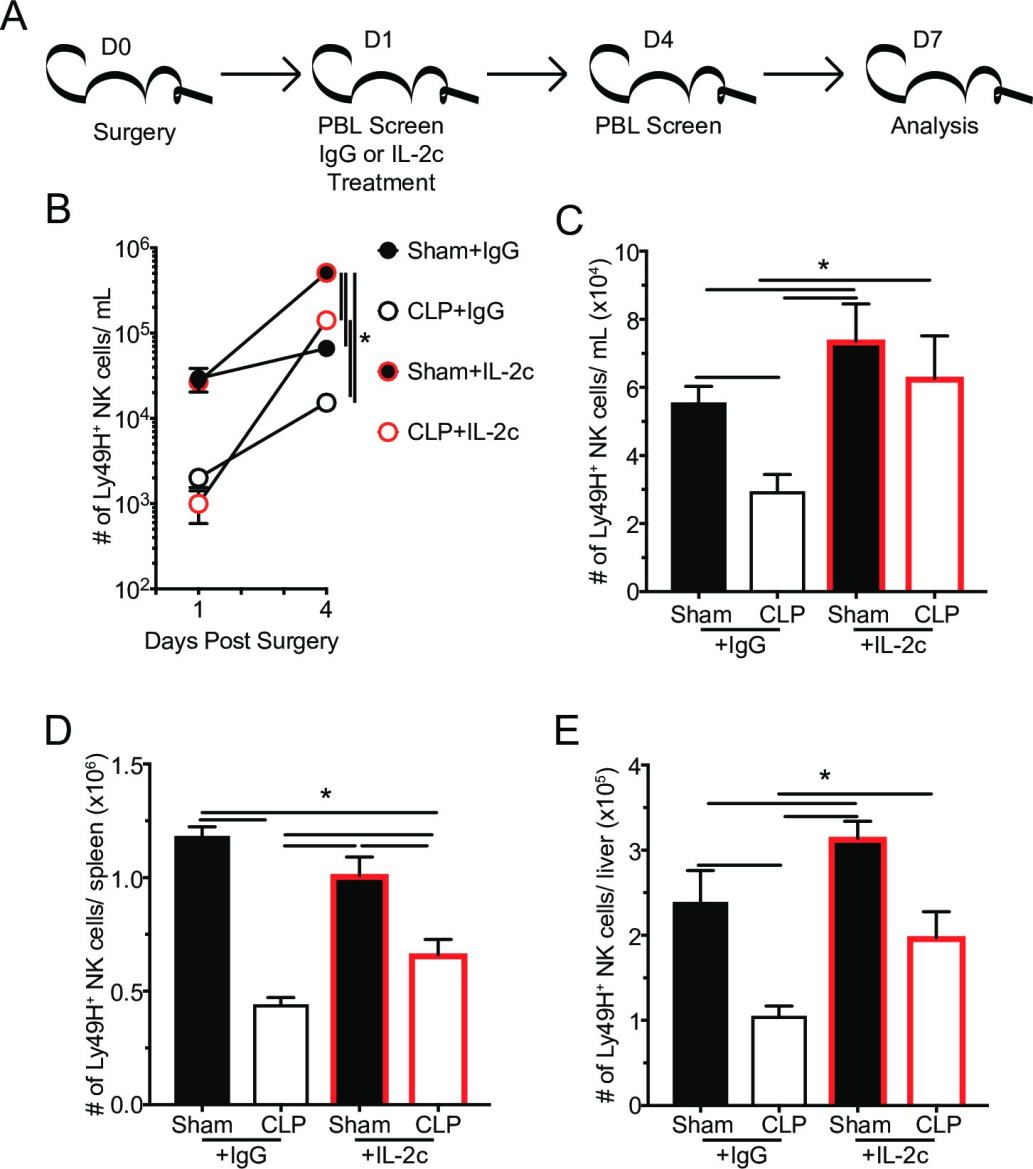
IL-2c therapy increases the number of Ly49H^+^ NK-cells. (A) Experimental Design. One day after surgery mice were treated with IL-2c or IgG (control). The number of Ly49H^+^ NK-cells in the PBL (B,C), spleens (D), and livers (E) of Sham and CLP mice at the indicated days post-surgery. Data are representative from 2 independent experiments with 4–5 mice per group. * p<0.05. Error bars represent the standard error of the mean.

The impaired control of MCMV infection highlights a need to recover the NK-cell compartment (number and/or function) in the post-septic environment. Recent clinical trials have demonstrated promise in recovering lymphocyte numbers by administration of lymphoproliferative cytokines (e.g. IL-2/7/15) [82–87]. Therefore, the efficacy of IL-2/α-IL-2 mAb complexes (IL-2c) therapy in reversing sepsis induced impairment of NK-cell-mediated MCMV control was explored next. The α-IL-2 mAb S4B6 was used for complex formation because it does not result in the expansion of regulatory T cells (T_reg_) [88, 89]. Mice received either control IgG or IL-2c at 24 hrs post-surgery, the earliest time point post-sepsis-induction at which the frequency of apoptotic cells in the spleen is no longer elevated (**Fig 1I**). Ly49H^+^ NK-cells in the PBL were monitored prior to therapy administration, 3 days after therapy (D4 post-sepsis), and 6 days after therapy (D7 post-sepsis). Spleen and liver Ly49H^+^ NK-cells were determined 6 days after therapy (**Fig 11A**). We observed robust expansion of Ly49H^+^ NK-cells in the PBL 3 days after IL-2c treatment in both groups of mice (**Fig 11B**). The number of Ly49H^+^ was also elevated in spleen, liver and PBL of IL-2c treated CLP hosts, relative to IgG treated controls 6 days post-treatment (**Fig 11C–11E**). It is, however, noteworthy that the number of Ly49H^+^ NK-cells in the spleen of IL-2c treated CLP hosts is still not fully recovered, relative to Sham mice. Thus, IL-2c therapy leads to robust expansion of Ly49H^+^ NK-cells in multiple tissues that remains elevated up to 6 days after surgery.

### IL-2c therapy does not improve DAP12 expression or intrinsic NK-cell function

**Fig 12:**
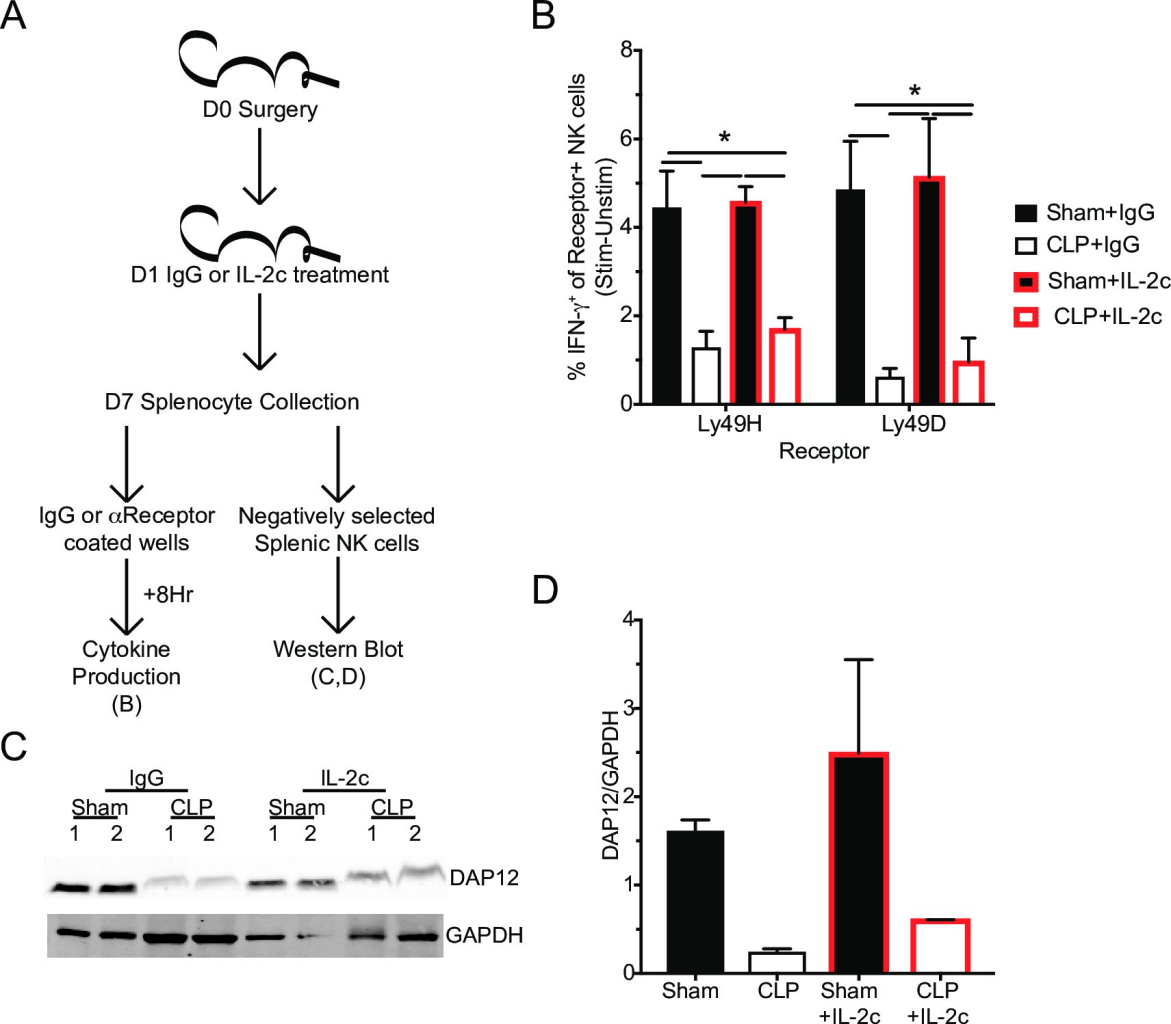
IL-2c therapy does not improve DAP12 expression or intrinsic NK-cell function. (A) Experimental Design. One day after surgery mice were treated with IL-2c or IgG (control). Cytokine production in response to receptor stimulation and DAP12 expression of NK-cells was determined 6 days later. (B) Frequency of IFN-**γ**^+^ of stimulated receptor^+^ NK-cells from Sham or CLP mice after 8 hrs stimulation with plate bound control (IgG), αLy49H, or αLy49D antibody. Immunoblot (C) and ratio quantification (D) for DAP12 and GAPDH in unstimulated NK-cells from Sham and CLP mice, numbers indicate replicates. Data are representative from 2 independent experiments with 4–5 mice per group in panel B and 1 independent experiment with 5x10^5^ cells per lane in panels C,D. * p<0.05. Error bars represent the standard error of the mean.

The numerical increase in Ly49H^+^ NK-cells in CLP host treated with IL-2c was interesting, but it was unclear whether this therapeutic intervention with IL-2c merely resulted in the proliferation of a ‘broken’ population of cells. Promisingly, IL-2 has been described to both increase the number of NK-cells and enhance activating receptor function (such as Ly49H), in part by tuning calcium flux [43, 58, 73, 90, 91, 92]. To address whether IL-2c therapy improved the intrinsic functional capacity of NK-cells, Sham and CLP mice were again treated with control IgG or IL-2c at D1 post-surgery. NK-cell capacity to produce IFN-**γ** in response to Ly49H or Ly49D receptor stimulation was evaluated as well as expression of DAP12 by NK-cells 6 days after start of the therapy (**Fig 12A**). The capacity of NK-cells from IgG-treated CLP hosts to produce IFN-**γ** in response to receptor stimulation remained impaired, demonstrating longevity of the previously described lesion. Additionally, NK-cells, from IL-2c treated CLP hosts, to produce IFN-**γ** in response to receptor stimulation also remained impaired, indicating that IL-2c does not improve the intrinsic function of NK-cells (**Fig 12B**). To determine whether this functional impairment (despite the numerical restoration) was associated with altered expression of DAP12, splenic NK-cells were isolated from Sham and CLP hosts 6 days after either IgG or IL-2c therapy (7 days post-surgery). Importantly, the reduced DAP12 expression observed in NK-cells from IgG-treated CLP hosts was not markedly increased upon IL-2c treatment further suggesting a strong association between DAP12 expression and diminished receptor-mediated cytokine production (**Fig 12C and 12D**). Thus, these results indicate impaired NK-cell receptor-mediated function is maintained after CLP induction and treatments that increase NK-cell numbers do not necessarily improve the ‘per-cell capacity’ of NK-cells to function properly.

### IL-2c therapy numerical recovery of Ly49H^+^ NK-cells partially rescues MCM viral control in CLP hosts

**Fig 13:**
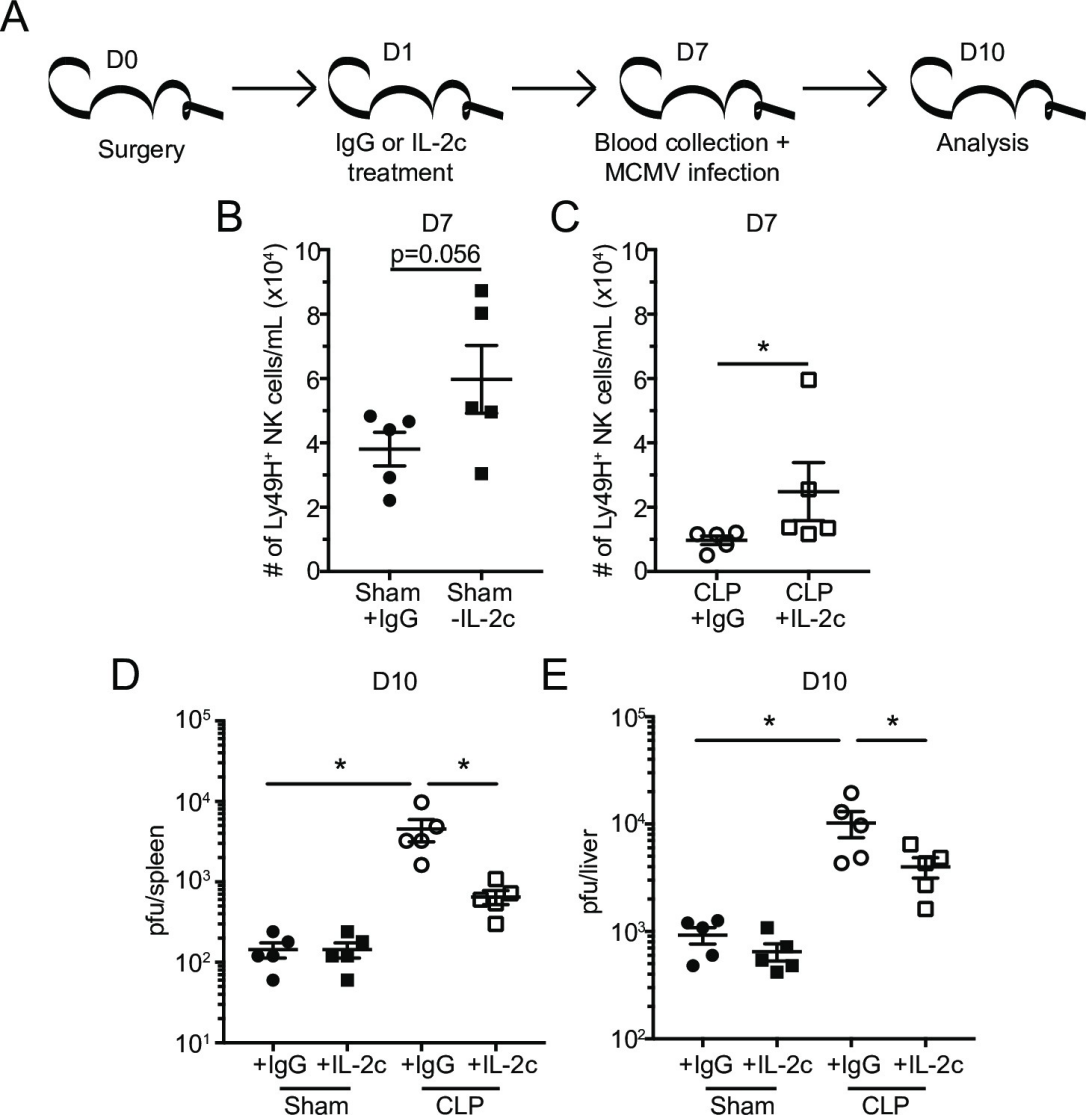
IL-2c therapy numerical recovery of Ly49H^+^ NK-cells partially rescues MCM viral control in CLP hosts. (A) Experimental Design. One day after surgery mice were treated with IL-2c or IgG (control) and infected with MCMV 6 days later. Number of Ly49H^+^ NK-cells in the PBL of Sham (B) and CLP (C) mice at the time of MCMV infection. Viral titers in the spleens (D) or livers (E) of Sham and CLP groups of mice. Data are representative from 3 independent experiments with 3–5 mice per group. * p<0.05. Error bars represent the standard error of the mean.

The results of **Figs 11** and **12** indicate that IL-2c rescues the number but not the intrinsic function of Ly49H^+^ NK-cells. However, we wanted to determine whether this numerical increase alone was sufficient to improve pathogen control. To address this, the numbers of Ly49H^+^ NK-cells were evaluated in the PBL 6 days after IL-2c therapy, a time at which cells are undergoing contraction following the IL-2-induced proliferative burst (**Fig 13A**) [93]. Importantly, IL-2c therapy induced an increase in Ly49H^+^ NK-cell numbers in PBL of both Sham and CLP mice at the time of MCMV challenge (Smith; 10^5^ PFU i.p) (**Fig 13B and 13C**). Viral titers were evaluated in the spleens and livers 3 days after infection (10 days post-surgery; **Fig 13D and 13E**). Viral titers in both the liver and spleens of Sham mice were low and indistinguishable indicating IL-2c therapy does not further improve control of MCMV in non-septic hosts (**Fig 13D and 13E**). IgG-treated CLP hosts had higher viral titers than their NK-sufficient Sham counterparts even 10 days post-surgery suggesting sepsis can induce long-term changes in NK-cells preventing them to function properly. Importantly, IL-2c-treated CLP mice showed statistically significant improvement in viral control (**Fig 13D and 13E**). Cumulatively, these data suggest that IL-2c therapy reduces sepsis-induced impairment of host NK-cell-mediated pathogen control potentially by improving the number but not the intrinsic function(s) of NK-cells.

## Discussion

Sepsis as systemic infection can lead to a cytokine storm causing tissue damage and/or death [1]. However, patient survival of the cytokine storm has gradually risen over the recent decades leading to a population of sepsis survivors [2, 3]. These sepsis survivors can, though, enter a state of chronic immunoparalysis characterized by increased susceptibility to pathogens and decreased long-term survival [4–6]. This immunoparalysis is defined by severe lymphopenia and reduced functionality of surviving lymphocytes [7–9, 31–41, 94]. Paradoxically, while NK-cells are beneficial during the early stages of infection, the contribution of NK-cells to the septic event, including cytokine release (e.g. IFN-**γ**), has largely framed these cells as detrimental in the context of sepsis [21–30]. Thus, their capacity to mediate early control of pathogens suggests sepsis-induced impairment of NK-cell function may also contribute to the immunoparalysis state of sepsis survivors. Indeed, their capacity to produce cytokine in response to infection and TLR stimulation is impaired, highlighting differential roles of NK-cells during the cytokine storm and sepsis-induced immunoparalysis [37, 38]. However, the scope of sepsis-induced lesions in NK-cells, the underlying cause of this functional loss, and its subsequent impact on host capacity to control infection had not been fully defined.

NK-cell response to target cells is dictated by a balance of activating and inhibitory receptor signaling [39–42]. Receptor expression and capacity to effectively signal will determine whether an immunologic synapse is formed with the target cell and effector function (e.g., cytokine production and cytolysis) is performed [78]. Thus, changes in the number of receptor expressing cells and/or their relative expression of the receptor or its capacity to signal can lead to changes in NK-cell capacity to mediate control of infections. Therefore, we investigated how sepsis impacts NK-cell receptor-mediated control of pathogens. Using Ly49H as a model receptor, we were able to effectively probe NK-cell-intrinsic impairments in receptor signaling, independent of receptor expression. This impairment, in conjunction with the sepsis-induced lymphopenic state led to a dramatic impairment in NK-cell-mediated pathogen control. Informatively, therapeutic administration of IL-2c led to the numerical increase in NK-cells and enhanced NK-cell-mediated pathogen control.

Our use of transcriptome analysis and GSEA provided a strong foundation for investigating how sepsis alters the function of NK-cells. The modulation in gene expression in NK-cells occurring within the first 2 days following sepsis induction was profound and served as the impetus for our interrogation of NK-cell effector function and Ly49H signaling. For example, the downregulation in genes associated with NK-cell cytotoxicity (e.g., *Ifng*, *Tnf*, and *Prf1*) correlated strongly with our data showing reduced frequencies of IFNγ^+^ NK-cells from septic hosts and reduced target cell killing. The reduced functionality connected with the revealed deficiencies in the expression of DAP12 and downstream signaling from the Ly49H receptor. Not surprisingly, the numerical and functional defects in NK-cells led to the marked reduction in NK-cell-specific protection against secondary pathogen challenge in the post-septic host. We chose MCMV as the model pathogen for testing the fitness of the NK-cell compartment, finding the impairment in NK-cell-mediated pathogen control lasted up to 10 days after the septic insult. It is unclear, however, how long this impairment in pathogen control lasts. The duration of the sepsis-induced immunoparalysis in preclinical and clinical setting remains difficult to reliably define and serves as a significant knowledge gap not only for NK-cells, but across other immune populations. This includes known impairments in dendritic cell (DC) cytokine production in the post-septic environment [19]. Loss of DC-derived cytokine may lead to NK-cell-extrinsic impairment in NK-cell function, similar to that observed in T cell function [19]. This combination of cell-intrinsic and extrinsic impairments is reminiscent of those observed in T cells [20].

The administration of exogenous cytokines can have a marked effect on the number and function of various immune cell populations in sepsis survivors, as demonstrated by the preclinical and clinical data touting the benefits of IL-2, IL-7, IL-15, and Flt3L (among others). Of all the common γ_c_ receptor binding cytokines, IL-2 is the only cytokine currently approved for medical use and most promising in terms of improving functional responses. IL-2 has been intensively studied and shown to lower the rate of opportunistic infections in AIDS patients [95] and reduce bacterial abscess after CLP surgery in mice [96]. IL-2, however, is highly toxic at clinically significant levels. IL-2 signaling via low-affinity receptors present in the endothelium promotes vascular leak syndrome (VLS) [97], leading to pulmonary edema, severe hypotension, and liver cell damage[98]. One way to avoid IL-2 toxicity (but maintain its potency) uses IL-2/α-IL-2 mAb complexes (IL-2c). IL-2 can signal via αβγ_c_ heterotrimers with high affinity, or βγ_c_ heterodimers with intermediate affinity. The action of IL-2 complexed to different α-IL-2 mAb, such as S4B6 IgG_1_ mAb, differs because these mAb can avoid interactions with low affinity receptors and bind a specific IL-2 receptor type. Thus, IL-2c (IL-2 bound by S4B6) expands effector cells (CD8 and CD4 T cells and NK-cells), and can lower pathogen burden in bacterial infections [99] without signs of VLS [100]. Thus, IL-2c therapy may serve as a short-term replacement in the absence of properly functioning DC (for example). This relates directly to the question of whether the supportive nature of the IL-2c therapy is sustained or wains with time. Understanding the duration of both the intrinsic/extrinsic impairment of NK-cells and therapeutic effect of IL-2c are important considerations that should be addressed in future experiments. While we observed that therapeutic IL-2c administration led to expansion of NK-cells and enhanced NK-cell-mediated pathogen control, it is clear that per-cell functional impairment still exists. This has direct relevance to recent assessment of therapeutic IL-7, a related cytokine, administration and indicates the need for additional therapeutic intervention beyond the numerical expansion that the study evaluated [82, 101].

Finally, a major consideration of the experiments performed and data presented is that, while intrinsic impairment in DAP12-dependent receptors, Ly49H and Ly49D, was observed it is not representative of all NK-cell receptors [55, 102]. Thus, it is important to address the impact of sepsis on other NK-cell receptors–for example, activating receptors such as NKG2D and NKG2C and inhibitory receptors such as NKG2A and PD-1 –in future experiments, in addition to NK1.1 and NKp46 assessed here. Evaluating other receptors, such as NKG2D, that can signal through DAP10, a related adaptor protein, may be of particular interest given that gene expression of DAP10 was not observed to be altered by sepsis [58]. Further determining how sepsis impacts the capacity of inhibitory receptors to limit activating receptors from signaling, by activating SHP-1 and SHP-2, may reveal additional nuances to impaired NK-cell signaling [103]. Sepsis increases the expression of PD-1 by NK-cells [104, 105]. This, and other similarly increased expression of inhibitory receptors, may contribute to shifting the receptor balance to an inhibitory state, in addition to the intrinsic impairment in activating receptor signaling, subsequently impairing NK-cell function. These data contribute to a growing body of literature on sepsis-induced lymphocyte intrinsic and extrinsic, which contribute to the immunoparalyzed state of sepsis survivors [20].

## Materials and methods

### Ethics statement

Experimental procedures using mice were approved by University of Iowa Animal Care and Use Committee under ACURF protocol numbers 7051102 and 6121915. The experiments performed followed Office of Laboratory Animal Welfare guidelines and PHS Policy on Humane Care and Use of Laboratory Animals. Cervical dislocation was used as euthanasia method of all experimental mice.

### Mice and pathogens

Inbred C57Bl/6 (B6; Thy1.2/1.2) and outbred Swiss Webster mice were purchased from the National Cancer Institute (Frederick, MD) and maintained in the animal facilities at the University of Iowa at the appropriate biosafety level. Perforin knockout mice (*Prf*^*-/-*^) were a generous gift from the Karandikar lab (Department of Pathology, University of Iowa). m157-transgenic (m157-Tg) mice (Thy1.2/Thy1.2) were obtained from the Tripathy lab at the Washington University (St. Louis) and were bred and maintained at the University of Iowa (Iowa City, IA). Recombinant virulent *Listeria monocytogenes* (*L*.*m*.; strain 1043S) was injected i.v. (10^4^ CFU). For MCMV-Smith infection, mice were injected i.p. (10^5^ PFU). MCMV viral titers were quantified using plaque assay on M2-10B4 cells, as previously described [81].

### Bone marrow chimeric mice

Bone marrow (BM) chimeric mice were generated as previously described [106]. Briefly, the *Tyrobp* gene was cloned into an overexpression plasmid (pMSCV-IRES-mCherry), gene insertion into plasmid was confirmed by sequencing. The *Tyrobp*-containing or control (ctrl) plasmid was then transfected into 293 cells before being infected with adenovirus to generate adenovirus constructs for BM progenitor transfection. Lin^-^ (B220, Gr-1, Ter119, NK1.1, TCRγ/δ, CD11b, CD11c, CD4, CD8, CD3) BM was collected from B6.SJL (CD45.1) mice and transfected with either of the adenoviral constructs. (5000 mCherry+ LSK cells along with 0.2 million protector BM cells) transfected BM was then injected into each irradiated 6–8 wk old C57Bl/6 recipients (CD45.2). Mice received Uniprim diet for the first 2 wks following transplant before returning to normal chow. 8 wks after transplantation mice were used for experiments.

### Cell isolation

Peripheral blood (PBL) was collected by retro-orbital bleeding. Peritoneal lavage was gathered by injecting 1mL of cold RPMI into the peritoneal cavity. The abdomen was then gently massaged before removing the fluid from the peritoneal cavity. Single-cell suspensions from spleen, liver, and lymph nodes were generated after mashing tissue through 70 μm cell strainer without enzymatic digestion. Livers were subsequently suspended in a Percoll (35%) and RPMI (65%) gradient to isolate mononuclear cells.

### Flow cytometry, peptides and cytokine detection

Flow cytometry data were acquired on a FACSCanto (BD Biosciences, San Diego, CA) and analyzed with FlowJo software (Tree Star, Ashland, OR). To determine expression of cell surface proteins, mAb were incubated at 4°C for 20–30 min and cells were fixed using Cytofix/Cytoperm Solution (BD Biosciences) and, in some instances followed by mAb incubation to detect intracellular proteins. The following mAb clones were used: NK1.1 (PK136, eBioscience), CD3 (17A2, eBioscience), Ly49H (3D10, eBioscience), Ly49D (4E5, eBioscience), NKp46 (29A1.4, eBioscience), CD27 (LG.7F9, eBioscience), CD11b (M1/70, eBioscience), IFN-γ (XMG1.2; eBioscience), Granzyme B (MHGB04, Invitrogen), CD107a (1D4B, BD Pharmingen), AKT1 (55/PKBa/AKT, BD Pharmigen), pS473 (M89-61, BD Pharmigen).

Intracellular cytokine staining: For direct *ex vivo*, staining cells were incubated for 1 additional hour in the presence of Brefeldin A (BFA) before surface and intracellular IFN-γ staining. For cytokine staining following *in vitro* stimulation BFA was added during the last hour of stimulation. Intracellular signaling staining: For detection of intracellular AKT and pAKT (pS473) cells were methanol fixed and permeabilized according to BD protocol. Apoptosis was evaluated using Vybrant FAM Caspase-3/7 Assay Kit (Invitrogen) according to manufacturer’s protocol.

### Assessment of *in vitro* cell viability

Splenocytes obtained from Sham or CLP hosts 48 hrs post-surgery were placed in culture for additional 48 hrs. At the end of each timepoint the number of live cells per well was enumerated and then assessed by flow cytometry to determine the frequency of live NK-cells. The % of input NK-cells was determined by the following equation: [(# of live NK-cells)_at indicated timepoint_] / [(# of live NK-cells)_prior to culture_].

### IL-12 and IL-18 stimulation

Splenocytes were incubated at 37°C with 20 ng/mL each of rIL-12 and rIL-18 (R&D Systems) for 8 hrs. BFA was added during the last 4 hrs of stimulation.

### CFSE cell labeling

Splenocytes (10^7^/mL) from m157 and wild type littermate control mice were labeled with CarboxyFluorescein diacetate Succinimidyl Ester (CFSE; eBioscience) by incubating the cells at room temperature for 15 minutes with 1μM (CFSE^hi^) or 0.1 μM (CFSE^lo^) CFSE. The labeled cells were incubated for 5 minutes with 1mL FCS on ice to remove any free CFSE, and washed three times with RPMI prior to adoptive transfer by i.v. injection, as we performed previously [107].

### M157 target cell killing assays

*In vivo* target cell killing was performed as previously described [66]. Briefly, target splenocytes from m157-Tg and littermate control mice were disparately labeled with CFSE (as described above). Target cells were mixed 1:1 before being injected (10^6^ total cells) i.v. into NK1.1-depleted and control (NK sufficient) Sham and CLP hosts and analyzed 3 hrs after injection. The ratio of CFSE^hi^:CFSE^lo^ cells was determined by flow cytometry. m157 specific lysis was calculated using the previously described calculation: [(1-(Ratio(CFSE^lo^: CFSE^hi^)_sample_/ Average(Ratio(CFSE^lo^: CFSE^hi^)_NK1.1-depleted_))) x 100]. Sham and CLP groups used separate NK1.1-depleted controls for corresponding group. The specific lysis calculation adjusts the killing for the different environments present in Sham and CLP hosts.

*In vitro* target cell killing used the same above target cell mixture. However, target cells in the absence of effector cells served as the control. Thus, the above equation was modified to be: [(1-(Ratio(CFSE^lo^: CFSE^hi^)_sample_/ Ratio(CFSE^lo^: CFSE^hi^)_target cells only_))) x 100]. Splenocytes were mixed with target cells at a ratio of 1:1:1 (splenocytes: m157: littermate), i.e. 0.5:1 (effector: target). Cells were incubated for 18 hrs at 37°C. Cells were surface stained to determine the number of Ly49H^+^ NK-cells present at the end of stimulation.

### M157 target cell degranulation assay

The same target cell mix, described above (Methods: M157 Target cell killing assays), was used. Splenocytes were stimulated at multiple effector-to-target cell ratios (sample w/m157 targets) in the presence of fluorescently labeled α-CD107a mAb and monensin for 6 hrs at 37°C. Splenocytes in the absence of target cells served as controls (sample w/WT targets). CD107a expression served as a marker of degranulation and was assessed by flow cytometry in conjunction with surface staining of NK-cells. Degranulation in response to receptor stimulation was calculated as (% of CD107a^+^ Ly49H^+^ NK-cells)_sample w/m157 targets_−(% of CD107a^+^ Ly49H^+^ NK-cells)_sample w/WT targets_

### Plate-bound antibody stimulation

Plate bound antibody stimulation was performed as described [73]. Briefly, flat-bottomed 96-well plates (Thermo-Fisher) were coated overnight (at 4°C) with 100μL of control IgG or fluorescently labeled anti-receptor antibody (diluted 1:100). Fluorescently-labeled antibody was used to identify cells that had internalized the receptor as a result of stimulation. Equivalent numbers of splenocytes were then stimulated for 8 hrs at 37°C in the presence of BfA to promote accumulation of cytokine. Cells were then surface stained, fixed/permeabilized, and stained intracellularly to evaluate cytokine production. Receptor specific response is calculated as % Stim[αreceptor antibody]- % Unstim[IgG] of receptor expressing cells.

### PMA and Ionomycin stimulation

Splenocytes were incubated at 37°C with 50μM phorbyl 12-myristate-13-acetate (PMA) and 500 μM Ionomycin (Iono) for 4 hrs. BFA was added during the last hour of stimulation.

### Calcium flux assay

Calcium flux assay was performed as described with some modification [108]. Briefly, 10^6^ splenocytes were labeled with Fluo-4AM according to manufacturer’s instructions (F10489, Thermo-Fisher). Cells were then surface stained. A baseline reading was taken for 20 seconds. Cells were then stimulated by adding fluorescently labeled α-Ly49H mAb and reading was taken for 110 seconds. Cells were finally stimulated with Iono (500μM) and readings were taken for 50 seconds.

### Immunoblotting

Immunoblotting was performed as described [108). Antibodies used for immunoblotting analysis were: monoclonal mouse/human α-DAP12 (D7G1X, Cell Signaling Technology) and mouse α-GAPDH (H86045M, Meridian Life Science). NK-cells were isolated by negative selection using Miltenyi Biotec NK-cell isolation kit (130-115-818) according to manufacturer’s instructions. 5x10^5^ cells were used for each well; Sham = 3 mice/sample, CLP = 9 mice/sample. Cells were then lysed with 2X lysis buffer (20mM Tris pH 8.0, 2mM EDTA, 2 mM Na_3_VO_4_, 20mM DTT, 2% SDS, and 20% glycerol) at 95°C for 5 min. Lysates were sonicated to reduce viscosity and loaded on 10–20% Tris-HCl Protein Gel (3450033, Bio Rad Criterion). Separated proteins were transferred to PVDF membranes (Millipore) and blocked for 1 hr in 1:1 PBS:SEA Block (Thermo-Fisher) IRDye 800CW or IRDye 680-conjugated secondary antibodies were diluted in SEA Block and incubated with PVDF membranes for 30 min at room temperature. Membranes were imaged using Licor Odyssey Infrared detector.

### TIRF microscopy

Images were taken using Leica AM TIRF MC imaging system as described with the following modifications [108]. NK-cells were isolated by negative selection and placed on glass chamber slides (5x10^4^ cells/chamber; LabTek II) precoated with 10μg/mL α-Ly49H mAb. Cells were stimulated for 15 minutes, fixed with 4% paraformaldehyde, and permeabilized with 0.25% Triton-X. Cells were blocked with SEA blocking buffer (Thermo-Fisher) for 1 hour and stained with 5 μL rabbit α-human/mouse DAP12 antibody (ab219765, Abcam) overnight at 4°C. Cells were washed and incubated with DyLight 488-conjugated donkey α-rabbit IgG (poly4064, BioLegend) secondary antibody for 2 hrs at room temperature. Cells were washed and fresh PBS was added to each well. Images were taken at room temperature using 100X oil submersion lens and Leica AM TIRF MC imaging system at the University of Iowa Central Microscopy Research Facility. Laser intensity and exposure parameters remained constant within each experiment. TIRF microscopy images were analyzed using ImageJ software. Membrane DAP12 was quantified by measuring mean pixel intensity in the longest axis of cells.

### Adhesion assay

Cellular adhesion was performed as previously described with some modification [108, 109]. Briefly, flat-bottomed 96-well plates (Thermo-Fischer) were coated with 0–10μg of αLy49H (3D10). 5x10^6^ splenocytes were incubated on the plate for 30 min. Non-adherent cells were removed by quickly inverting the plate to empty contents. Adherent cells were stained with αNK1.1-APC-Cy7 (PK136). Cells were washed twice with PBS before being imaged utilizing Licor Odyssey Infrared detector.

### Cecal ligation and puncture (CLP) model of sepsis induction

Mice were anesthetized with ketamine/xylazine (University of Iowa, Office of Animal Resources), the abdomen was shaved and disinfected with Betadine (Purdue Products), and a midline incision was made. The distal third of the cecum was ligated with Perma-Hand Silk (Ethicon), punctured once using a 25-gauge needle, and a small amount of fecal matter extruded. The cecum was returned to abdomen, the peritoneum was closed with 641G Perma-Hand Silk (Ethicon), and skin sealed using surgical Vetbond (3M). Following surgery, 1 mL PBS was administered s.c. to provide post-surgery fluid resuscitation. Bupivacaine (Hospira) was administered at the incision site, and flunixin meglumine (Phoenix) was administered for postoperative analgesia. This procedure created a septic state characterized by loss of appetite and body weight, ruffled hair, shivering, diarrhea, and/or periorbital exudates with 0–10% mortality rate, similar to our previous reports [16–19]. Sham mice underwent identical surgery excluding cecal ligation and puncture.

### RNA sequencing and gene set enrichment analysis

Total RNA was extracted from NK1.1^+^CD3^-^ cells sorted 1-day post-CLP and 2 days post-Sham or CLP, two biological replicates were obtained for each group. RNA-seq. was performed as previously described and was processed by the University of Iowa Bioinformatics Division [110]. Gene expression is given as DESEQ2 values. The sequencing quality of RNA-seq. libraries was assessed by FastQC v0.10.1 (http://www.bioinformatics.babraham.ac.uk/projects/fastqc/). RNA-seq. libraries were mapped to mouse genome using Tophat (v2.1.0) [111], and gene expressions were calculated with featureCounts, a read summarization program suitable for counting reads generated from RNA-seq. [112]. The reproducibility of RNA-seq. data was evaluated and visualized by PCA and correlation heatmap [113]. Pair-wise group comparative analyses were performed with DESeq2 with multiple test correction of fdr [114] to identify differentially expressed genes. Upregulated or downregulated genes in when comparing groups were identified by requiring a greater than 1.5-fold expression change and a false discovery rate (FDR) <0.05, as well as a non-zero DESEQ2 value. UCSC genes from the iGenome mouse mm9 assembly (https://support.illumina.com/sequencing/sequencing_software/igenome.html) were used for gene annotation. The RNA-seq. data are deposited at the GEO (accession number GSE#114739). Principal component analysis was performed using MATLAB R2017a software. Gene set enrichment and functional assignment were performed in DAVID bioinformatics resources and software from the Broad Institute as described [107, 110, 115]. Enrichment was evaluated for Day 2 CLP samples relative to Day 2 SHAM samples.

### IL-2/anti-IL-2 mAb complexes (IL-2c)

Complexes were made as previously described [89, 116]. Briefly, murine IL-2 (PeproTech) was incubated with S4B6 α-IL-2 mAb at a 2:1 molar ratio (1.5μg/mL IL-2: 50μg/mL S4B6) at 37°C for 15 min. 1.5μg of rat IgG or murine IL-2/IL-2c were injected.

### Statistical analysis

Unless stated otherwise data were analyzed using Prism6 software (GraphPad) using two-tailed Student t-test (for 2 individual groups, if unequal variance Mann-Whitney U test was used), one-way ANOVA with Bonferroni post-hoc test (for >2 individual groups, if unequal variance Kruskal-Wallis with Dunn’s post-hoc test was used), two-way ANOVA (for multiparametric analysis of 2 or more individual groups, pairing was used for samples that came from the same animal) with a confidence interval of >95% to determine significance (*p ≤ 0.05). Data are presented as standard error of the mean.

## Supporting information

S1 Fig Fratricide is not required for NK-cell loss during sepsis. (A) Experimental Design. 1 day prior to surgery (D-1) Thy1.2 WT and *Prf*^*-/-*^ mice received 5x10^6^ splenocytes from naïve Thy1.1^+^ WT donor mice. The number of Thy1.1^+^ donor NK-cells per spleen of recipient mice was determined 2 days after surgery. (B) Representative gating of Thy1.1^+^ NK-cells in recipient spleens. (C) The number of donor NK-cells in WT and *Prf*^*-/-*^ recipient spleens following Sham or CLP surgery. (D) Fold loss of CLP donor NK-cells relative to the average number of NK-cells recovered from Sham mice for respective recipient. Data are representative from 2 independent experiments with 3–5 mice per group. Numbers above bars show fold change between groups. * p<0.05. Error bars represent the standard error of the mean.
(TIF)

S2 Fig Sepsis results in numerical loss of NK-cells in outbred mice. (A) Experimental Design. 2 days after surgery outbred Swiss Webster (SW) the number of NK-cells in the liver and spleen was determined. (B) Representative flow plots of NK-cell gating. The number of NK-cells in the spleen (C) or liver (D). Data are representative from 2 independent experiments with 3–5 mice per group. Numbers above bars show fold change between groups. * p<0.05. Error bars represent the standard error of the mean.
(TIF)

S3 Fig Sepsis does not alter the maturation status of Ly49H^+^ NK-cells *in vivo*. **A)** Representative flow plots of NK-cell populations as defined by CD27 and CD11b, in both the spleen and liver 2 days after Sham or CLP surgery. The frequency or number of Ly49H^+^ NK-cell maturation populations in the spleen (B,D) or liver (C,E). Frequency and representative flow plots of Ly6C^+^ (F) and KLRG1^+^ (G) Ly49H^+^ NK-cells. (H) Representative flow plots. The GMFI of GzmB in Ly49H^+^ NK-cells in spleen (I) or liver (J). Data are representative from 3 independent experiments with 3–5 mice per group. * p<0.05. Error bars represent the standard error of the mean.
(TIF)

S4 Fig Sepsis impairs NK-cell receptor-mediated adherence. (A) Representative images of adherence NK-cells to αLy49H coated plates. (B) Quantification of Sham and CLP NK-cell adherence to plates at indicated concentration of αLy49H antibody. Data are representative from 1 independent experiment with 4–5 mice per group. * p<0.05. Error bars represent the standard error of the mean.
(TIF)

S5 Fig Sepsis partially influences NK-cell capacity to produce IFN-γ in response to PMA/Ionomycin stimulation. (A) Experimental Design. Splenocytes were obtained 2 days after surgery and IFN-γ production determined after 6 hrs of *in vitro* stimulation with PMA/Ionomycin. (B) Representative flow plots of IFN-γ producing NK-cells (total or Ly49H subset). The frequency of IFN-γ^+^ NK-cells in the spleen (C) or liver (D). Data are representative from 4 independent experiments with 3–5 mice per group. * p<0.05. Error bars represent the standard error of the mean.
(TIF)
