# Supplementary figures and images for: Polymicrobial sepsis influences NK-cell-mediated immunity by diminishing NK-cell-intrinsic receptor-mediated effector responses to viral ligands or infections
Source: PLoS Pathog. 2018 Oct 31;14(10):e1007405. doi: 10.1371/journal.ppat.1007405 (PMC6231673; doi:10.1371/journal.ppat.1007405)

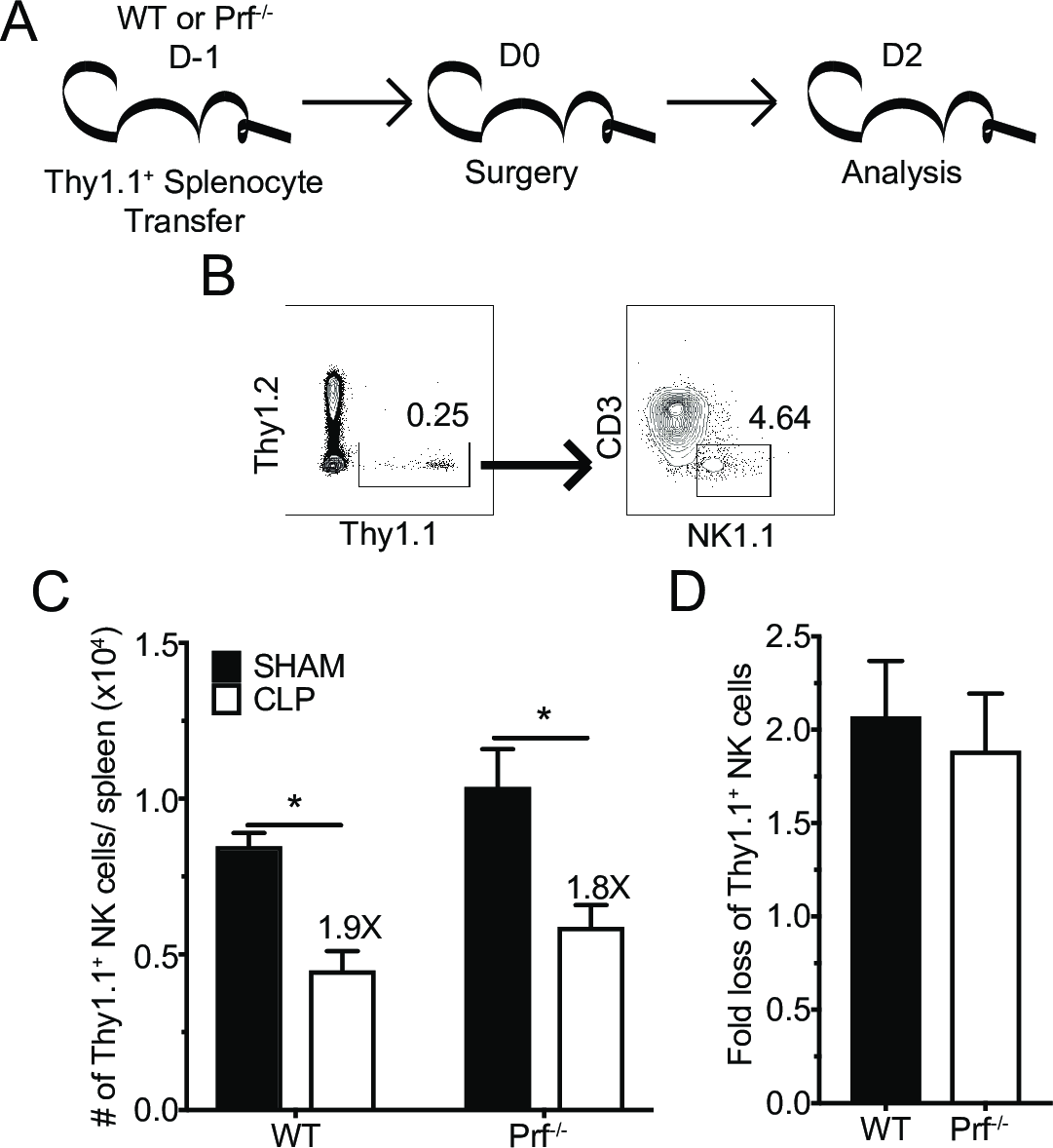

Supplement: S1 Fig — (A) Experimental Design. 1 day prior to surgery (D-1) Thy1.2 WT and Prf-/- mice received 5x10^6^ splenocytes from naïve Thy1.1+ WT donor mice. The number of Thy1.1+ donor NK-cells per spleen of recipient mice was determined 2 days after surgery. (B) Representative gating of Thy1.1+ NK-cells in recipient spleens. (C) The number of donor NK-cells in WT and Prf-/- recipient spleens following Sham or CLP surgery. (D) Fold loss of CLP donor NK-cells relative to the average number of NK-cells recovered from Sham mice for respective recipient. Data are representative from 2 independent experiments with 3–5 mice per group. Numbers above bars show fold change between groups. * p<0.05. Error bars represent the standard error of the mean. (TIF) [file ppat.1007405.s001.tif]

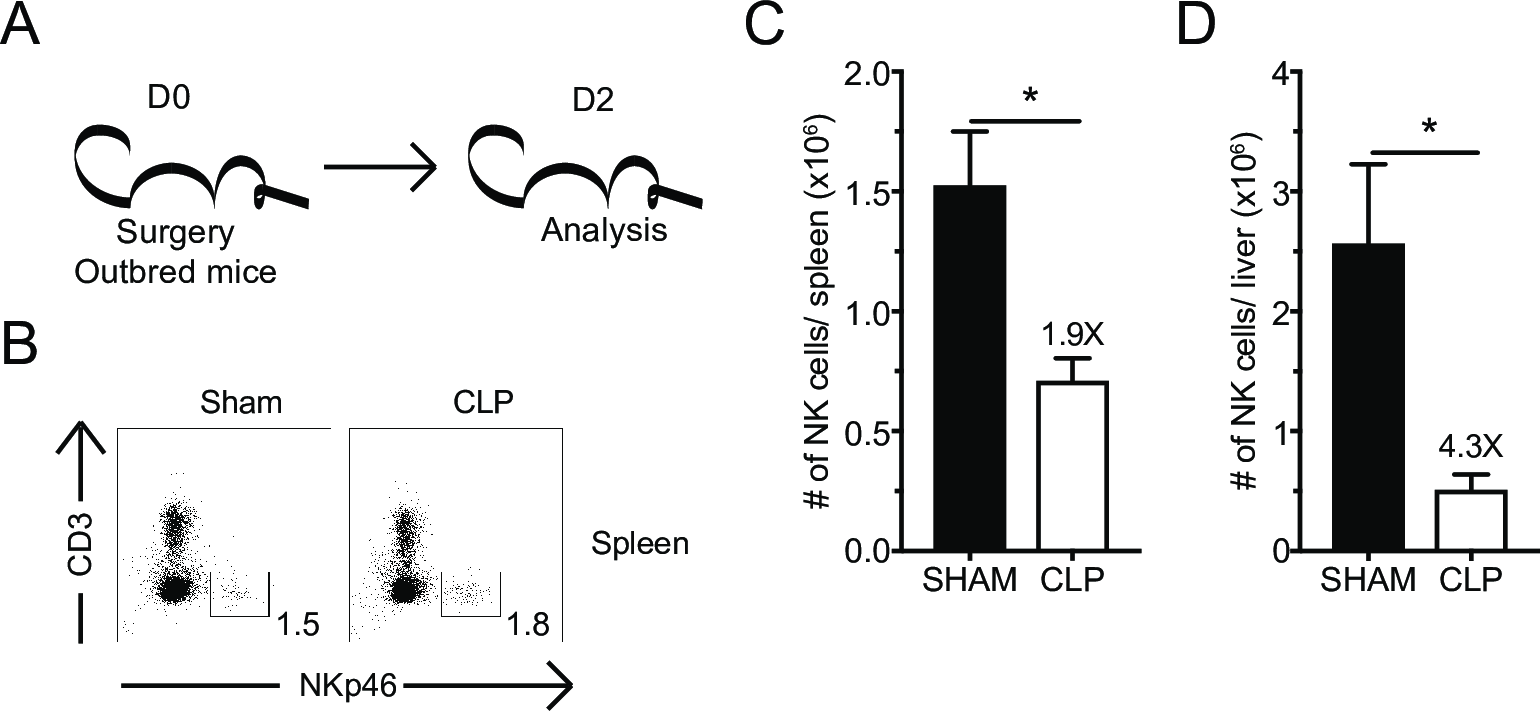

Supplement: S2 Fig — (A) Experimental Design. 2 days after surgery outbred Swiss Webster (SW) the number of NK-cells in the liver and spleen was determined. (B) Representative flow plots of NK-cell gating. The number of NK-cells in the spleen (C) or liver (D). Data are representative from 2 independent experiments with 3–5 mice per group. Numbers above bars show fold change between groups. * p<0.05. Error bars represent the standard error of the mean. (TIF) [file ppat.1007405.s002.tif]

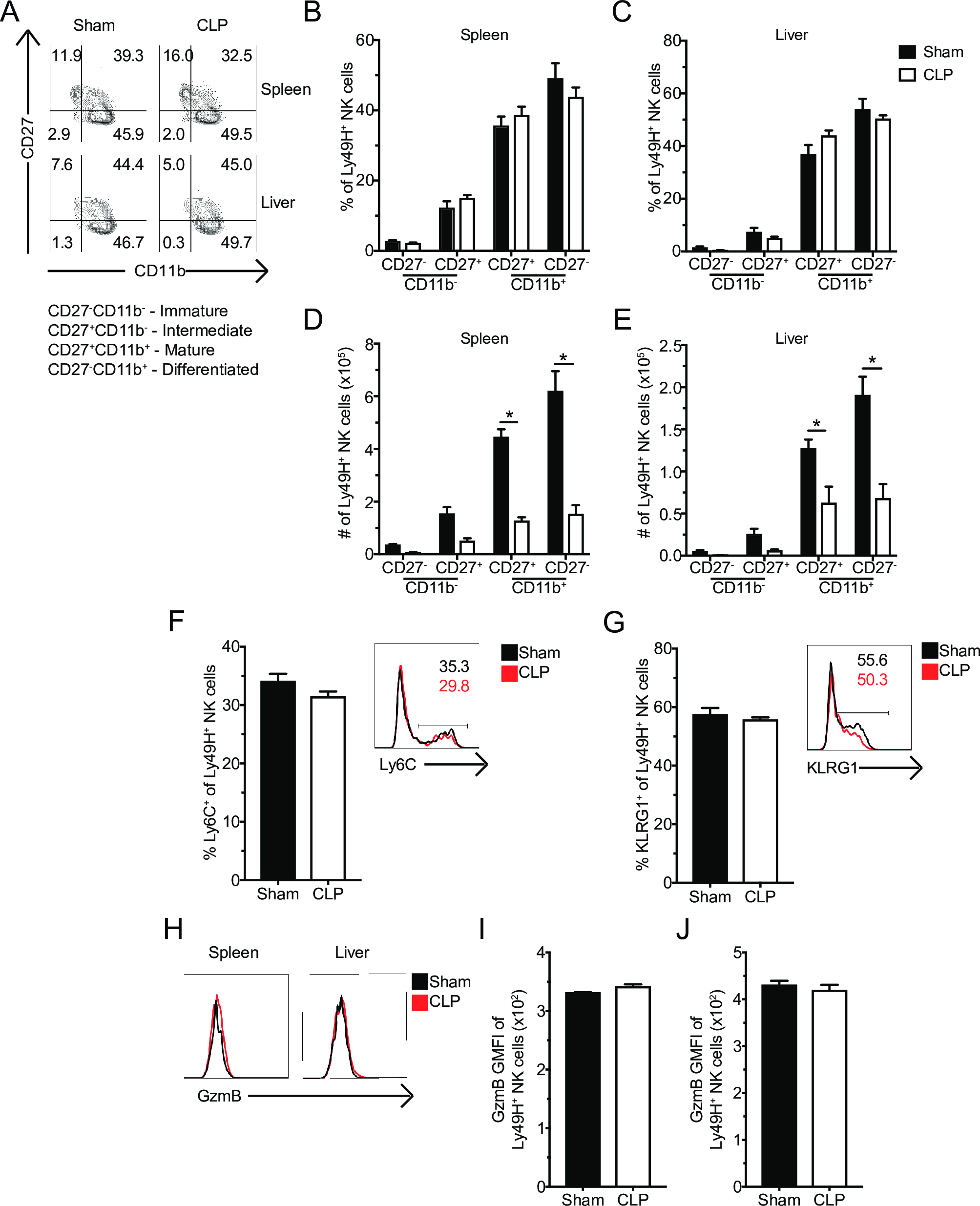

Supplement: S3 Fig — A) Representative flow plots of NK-cell populations as defined by CD27 and CD11b, in both the spleen and liver 2 days after Sham or CLP surgery. The frequency or number of Ly49H+ NK-cell maturation populations in the spleen (B,D) or liver (C,E). Frequency and representative flow plots of Ly6C+ (F) and KLRG1+ (G) Ly49H+ NK-cells. (H) Representative flow plots. The GMFI of GzmB in Ly49H+ NK-cells in spleen (I) or liver (J). Data are representative from 3 independent experiments with 3–5 mice per group. * p<0.05. Error bars represent the standard error of the mean. (TIF) [file ppat.1007405.s003.tif]

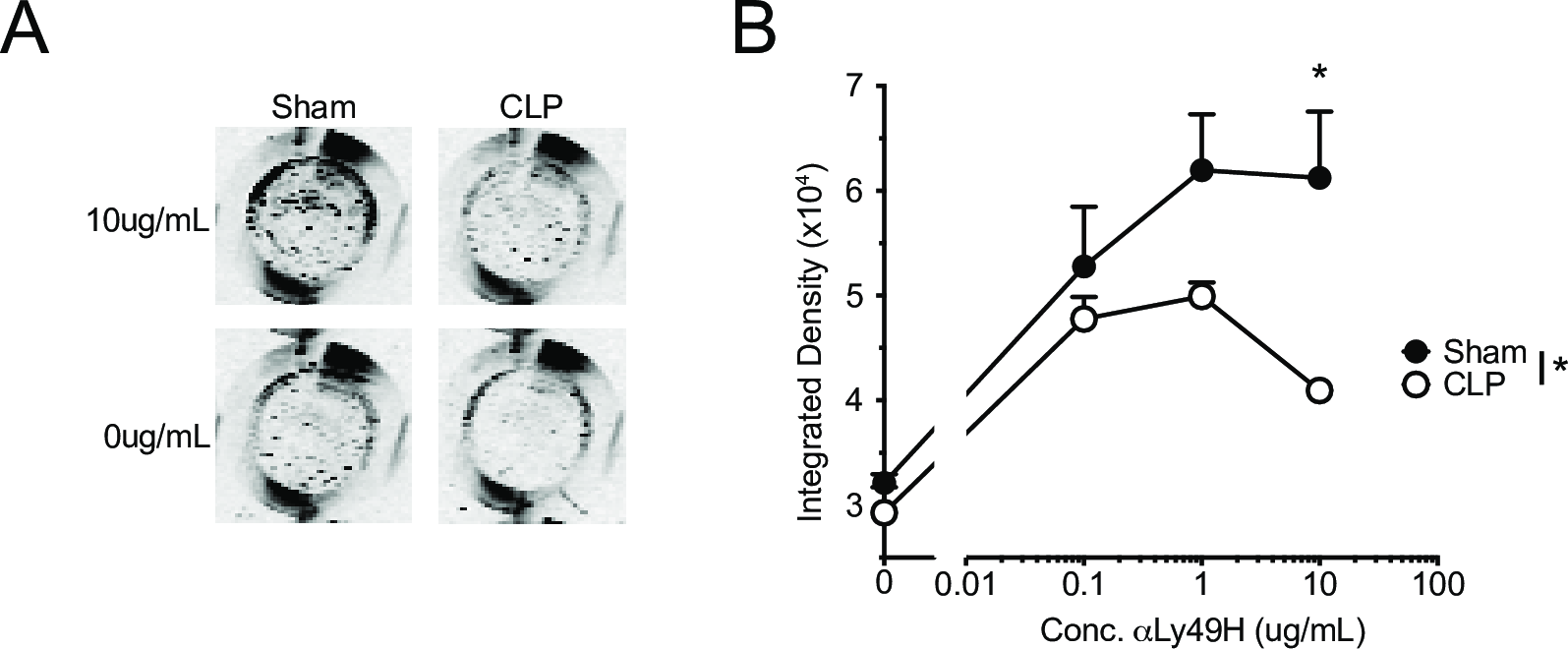

Supplement: S4 Fig — (A) Representative images of adherence NK-cells to αLy49H coated plates. (B) Quantification of Sham and CLP NK-cell adherence to plates at indicated concentration of αLy49H antibody. Data are representative from 1 independent experiment with 4–5 mice per group. * p<0.05. Error bars represent the standard error of the mean. (TIF) [file ppat.1007405.s004.tif]

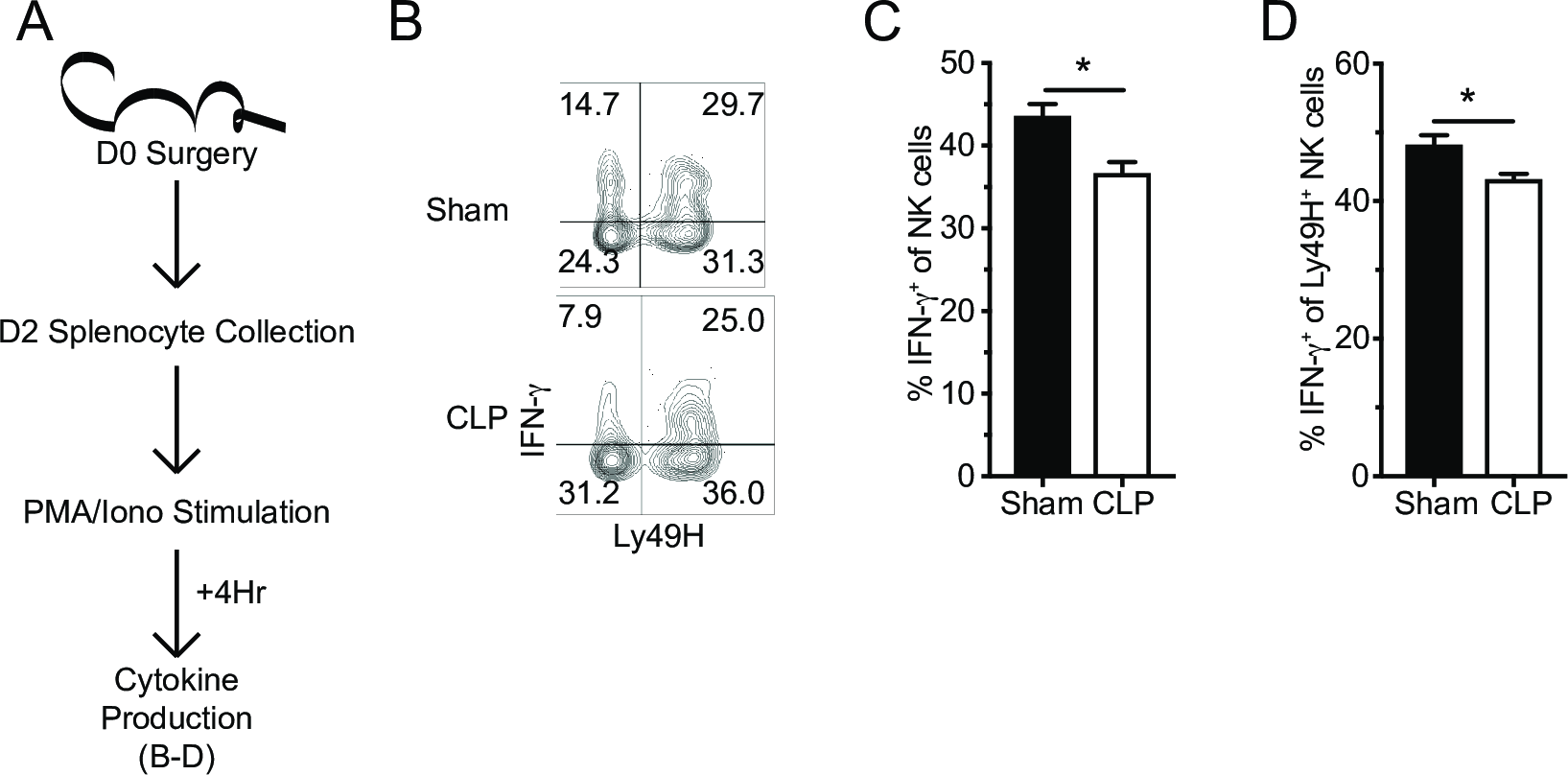

Supplement: S5 Fig — (A) Experimental Design. Splenocytes were obtained 2 days after surgery and IFN-γ production determined after 6 hrs of in vitro stimulation with PMA/Ionomycin. (B) Representative flow plots of IFN-γ producing NK-cells (total or Ly49H subset). The frequency of IFN-γ+ NK-cells in the spleen (C) or liver (D). Data are representative from 4 independent experiments with 3–5 mice per group. * p<0.05. Error bars represent the standard error of the mean. (TIF) [file ppat.1007405.s005.tif]
